# Stage-Specific lncRNA–mRNA Co-Expression Networks in Chicken Granulosa Cells Across Hierarchical Follicle Development

**DOI:** 10.3390/ani16091351

**Published:** 2026-04-28

**Authors:** Liang Li, Xue Han, Lulin Tan, Ya Tan, Lili Zhu, Yilong Li, Lin Luo, Jiahai Wu

**Affiliations:** 1 Institute of Animal Husbandry and Veterinary, Guizhou Academy of Agricultural Science, Guiyang 550005, Chinatanya_lee@126.com (Y.T.); 17585258762@163.com (Y.L.); luolin20001126@163.com (L.L.); jhw200609@163.com (J.W.); 2 Guizhou Provincial Key Laboratory of Livestock and Poultry Genetic Resources Innovation and Utilization, Guiyang 550005, China; 3 Guizhou Provincial Grassland Technology Test and Extension Station, Guiyang 550001, China; zhuerms@163.com; 4 Key Laboratory of Crop Genetic Resources and Germplasm Innovation in Karst Region, Ministry of Agriculture and Rural Affairs, Guiyang 550006, China

**Keywords:** chicken, granulosa cell, lncRNA, follicle hierarchy, ovulation

## Abstract

Long non-coding RNAs (lncRNAs) regulate granulosa cell function, but their stage-specific dynamics across the chicken follicle hierarchy remain unclear under matched endocrine conditions. We performed Ribo-Zero RNA sequencing on granulosa cells from small yellow follicles (SYF), F5, F2, and F1 follicles collected at the preovulatory luteinizing hormone (LH) surge. A multi-predictor pipeline (CPC2, CNCI, CPAT, PfamScan) identified 26,923 stringently filtered lncRNAs together with 15,838 mRNAs. Consecutive stage comparisons detected 2094, 1085, and 4318 differentially expressed genes and 671, 267, and 2762 differentially expressed lncRNAs in F5 vs. SYF, F2 vs. F5, and F1 vs. F2, respectively, with the most extensive remodeling at the F2-to-F1 transition. The F1 vs. SYF contrast captured the cumulative transcriptional difference across the hierarchy. Enrichment and temporal clustering showed that early hierarchical stages were characterized by proliferative, metabolic, and steroidogenic programs, whereas F1 granulosa cells were enriched for extracellular-matrix remodeling, MAPK signaling, and calcium ion binding. Weighted gene co-expression network analysis identified 10 stage-associated modules and highlighted candidate lncRNAs linked to lipid metabolism, angiogenesis, extracellular-matrix remodeling, and DNA repair, including *G5825*–*MYLIP*, *G66587*–*VEGFA*, and *G60212*–*CKS1B*. qPCR validation confirmed concordant expression trends for eight representative pairs. These results define a stage-resolved lncRNA–mRNA landscape across chicken follicle development and provide candidates for mechanistic studies of follicle maturation and periovulatory remodeling.

## Simple Summary

The hen ovary contains follicles at different stages of development at the same time, and the surrounding granulosa cells change their activity as each follicle matures. Whether long non-coding RNAs follow the same stage-dependent pattern in chickens has remained unclear. We analyzed granulosa cells from small yellow follicles and three progressively larger preovulatory follicles collected during the same preovulatory hormone surge and found marked changes in both long non-coding and protein-coding transcripts across the follicular hierarchy. Network analysis linked several transcript pairs to lipid metabolism, blood-vessel development, tissue remodeling, and DNA repair. Because the four follicle stages were sampled within the same LH-surge window, these transcript changes could be interpreted in relation to defined positions in the follicular hierarchy. These results provide a clearer picture of gene regulation during follicle maturation and identify candidate molecules for future studies on hen reproduction.

## 1. Introduction

In laying hens, a preovulatory luteinizing hormone (LH) surge drives ovulation at roughly 24 h intervals and peaks 4–6 h before follicle rupture [1]. Ovarian follicles are arranged in a clear size-based hierarchy: prehierarchical small yellow follicles (SYF, 6–8 mm) enter the preovulatory cohort and subsequently advance from F5 to F1 (10–40 mm) [2,3]. The four sampled classes represented the prehierarchical stage and early, middle, and immediately preovulatory positions within the hierarchical series. Because these follicle stages coexist within a single ovulatory cycle while occupying consecutive maturational states, the hen ovary provides a tractable system for dissecting stage-specific gene regulation under a shared hormonal background.

Granulosa cell differentiation depends on sequential gonadotropin signaling. FSH promotes recruitment of prehierarchical follicles by inducing FSHR expression [2,4]. Li et al. [5] used ONT long-read sequencing to show that follicle selection is accompanied by transcriptomic changes, including differentially expressed DHCR7 transcript variants linked to cholesterol and steroid biosynthesis. In hierarchical follicles, acquisition of LH responsiveness through *LHCGR* is a central feature of terminal granulosa cell differentiation [2]. As hierarchical follicles advance toward ovulation, programs governing proliferation, steroidogenesis, and endocrine responsiveness are progressively remodeled [6,7]. ERK1/2-linked signaling has been implicated in lipogenesis and steroidogenesis in chicken granulosa cells [8], and matrix metalloproteinase activity together with vascular reorganization are characteristic of the periovulatory transition [9,10]. The net effect of these sequential adjustments is a shift from a growth-promoting state toward terminal differentiation and periovulatory competence.

Long non-coding RNAs (lncRNAs)—transcripts exceeding 200 nucleotides and lacking protein-coding capacity—have emerged as regulators of granulosa cell biology in both mammalian and avian systems [11,12]. In mammalian models, individual lncRNAs have been functionally tied to granulosa cell apoptosis, autophagy, steroidogenesis, and proliferation [13,14]. For the chicken, long-read sequencing and chromatin-accessibility profiling refined the catalog of coding and non-coding transcripts that shift at follicle selection [15,16], and bulk RNA-seq profiling subsequently described broader transcriptome changes during follicle maturation [6,7]. Single-cell analyses further resolved stage-specific ovarian cell populations and uncovered transcriptional heterogeneity not visible in bulk data [17,18]. Specific chicken lncRNAs have been implicated in granulosa cell proliferation, apoptosis, and steroidogenesis [19], and systems-level transcriptomic surveys of the hypothalamic–pituitary–ovarian axis have mapped the endocrine setting that precedes hierarchical follicle entry [20,21]. Despite this progress, no study has simultaneously profiled lncRNA and mRNA programs across SYF, F5, F2, and F1 granulosa cells isolated from the same preovulatory endocrine window, leaving the stage at which non-coding transcription is most extensively remodeled during the hierarchical series unresolved.

We therefore performed Ribo-Zero RNA sequencing on granulosa cells from prehierarchical (SYF) and hierarchical (F5, F2, F1) follicles collected at the LH surge. By integrating consecutive-stage and hierarchy-spanning differential-expression contrasts with cis/trans-target prediction, WGCNA, and qPCR validation, this study mapped stepwise changes in coding and non-coding transcriptional programs across the chicken follicular hierarchy.

## 2. Materials and Methods

### 2.1. Ethics Approval

All animal procedures were approved by the Animal Welfare and Ethics Committee of the Guizhou Provincial Institute of Animal Husbandry and Veterinary (approval No. AWE-GZSXMSY-2025-02) and complied with institutional guidelines. Lohmann Pink-shell layers were maintained under standard conditions (16 h light: 8 h dark; temperature-controlled house) with feed and water provided ad libitum.

### 2.2. Animals, LH Profiling, and Histology

Three hundred healthy Lohmann layers (120 days old) were housed under a long-day photoperiod (16 h light: 8 h dark; lights on at 06:00). LH profiling was performed across a full 24 h cycle, and transcriptomic sampling was anchored to the observed serum LH peak. Oviposition times were recorded daily to estimate ovulation timing. At 210 days of age, birds that consistently laid eggs at approximately 08:00 were selected. Blood samples were collected from the wing vein at six time points across a 24 h cycle (08:00, 12:00, 16:00, 20:00, 00:00, and 04:00; *n* = 6 per time point). Serum was separated by centrifugation (3000× *g*, 10 min, 4 °C) and stored at −80 °C. Serum LH concentrations were measured with a chicken LH ELISA kit (Cat. No. JL15950, Jianglai Biotech, Shanghai, China) following the manufacturer’s protocol. All samples were diluted 2-fold with the kit’s universal diluent before loading. Optical density was read at 450 nm on a microplate reader, and concentrations were calculated from a four-parameter logistic (4PL) standard curve fitted to the kit’s reference standards (0.312–20 ng/mL) and multiplied by the dilution factor. The LH-profiling birds and RNA-seq birds were separate individuals from the same experimental cohort and were maintained under identical housing and photoperiod conditions. For RNA-seq, three birds collected at the LH peak (04:00) served as biological replicates. Granulosa layers from SYF, F5, F2, and F1 follicles were isolated separately from each bird, snap-frozen in liquid nitrogen, and stored at −80 °C until RNA extraction. Within each bird, granulosa layers from 3 to 5 SYF (6–8 mm diameter) were pooled, whereas F5, F2, and F1 samples each represented a single follicle. For histology, one follicle per stage was collected at 04:00 from separate birds in the same experimental cohort; for SYF, the largest follicle within the 6–8 mm SYF class was selected for H&E staining. Samples were fixed in 4% paraformaldehyde, embedded in paraffin, and stained with hematoxylin and eosin (H&E). Histological and RNA-seq samples were therefore obtained from different individuals.

### 2.3. RNA Isolation, Library Construction, and Sequencing

Total RNA was extracted with TRIzol reagent (Invitrogen, Carlsbad, CA, USA) according to the manufacturer’s instructions. RNA integrity and concentration were assessed on an Agilent 2100 Bioanalyzer (Agilent Technologies, Palo Alto, CA, USA) and a NanoDrop spectrophotometer (Thermo Fisher Scientific, Wilmington, DE, USA). Strand-specific libraries were prepared with the Ribo-Zero™ Gold Kit (Illumina, San Diego, CA, USA) and sequenced on an Illumina NovaSeq platform at Novogene Corporation (Beijing, China). Raw sequencing data were deposited in the BioProject database at the National Genomics Data Center (NGDC), China National Center for Bioinformation, under accession number PRJCA060696.

### 2.4. Transcriptome Mapping, lncRNA Identification, Quantification, and Classification

Clean reads were aligned to the chicken reference genome with STAR (v.2.6.0c). Transcripts were assembled by StringTie (v.2.1.1), and those shorter than 200 bp or containing a single exon were removed; the remaining assemblies were merged with TACO (v.0.7.3). Protein-coding potential was assessed by CPC2 (Coding Potential Calculator v.2), CNCI (Coding-Non-Coding Index, v.2), and CPAT (Coding-Potential Assessment Tool, v.3.0.4). EMBOSS Transeq translated the putative non-coding transcripts into all six reading frames, and PfamScan (v.1.6) screened the translations against Pfam (v.31) to exclude sequences harboring known protein domains. Only transcripts classified as non-coding by all four tools were retained as high-confidence lncRNAs. Expression levels (TPM) of mRNAs and lncRNAs were quantified with Kallisto (v.0.51.1), and lncRNAs were classified by genomic context (e.g., intergenic, intronic, sense, antisense) using the FEELnc (v.0.2) classifier.

### 2.5. Differential Expression Analysis

Differential expression of mRNAs and lncRNAs between consecutive stages was tested with DESeq2 (v1.16.1) [22] using raw read counts as input. For this design with three biological replicates per stage, DESeq2 estimated gene-wise dispersions by sharing information across genes, and Benjamini–Hochberg adjustment was used to control the false discovery rate. Genes with |log_2_(fold change)| > 1 and adjusted *p* < 0.05 were classified as differentially expressed. TPM values were used for expression filtering and visualization: transcripts with TPM > 0.1 in at least one library were considered expressed and retained for PCA and pairwise Pearson correlation analysis.

### 2.6. Functional Enrichment Analysis of Putative lncRNA Targets

Both cis- and trans-regulatory targets were predicted for differentially expressed lncRNAs. For cis-regulation, DEGs located within 100 kb upstream or downstream of a DEL were considered candidate targets. Pearson’s correlation coefficients between the expression profiles of DELs and neighboring DEGs were computed with the Hmisc R package (v.4.5); pairs meeting |r| > 0.95 and *p* < 0.05 were retained. For trans-regulation, genome-wide Pearson correlations between all DEL–DEG expression pairs were calculated; pairs with |r| ≥ 0.9 and Benjamini–Hochberg-adjusted *p* < 0.05 were kept. The resulting cis- and trans-target gene sets were subjected to Gene Ontology (GO) and Kyoto Encyclopedia of Genes and Genomes (KEGG) enrichment analyses on the KOBAS platform (v.3.0.3, Docker local deployment; http://bioinfo.org/kobas). GO terms and KEGG pathways with adjusted *p* < 0.05 were considered significant. The 100 kb window was used to define putative cis-regulation, consistent with previous lncRNA target-prediction analyses in chicken granulosa cells. Correlation cutoffs were set at |r| > 0.95 for cis pairs and |r| ≥ 0.9 for trans pairs to retain strongly co-expressed DEL–DEG associations.

### 2.7. Gene Expression Profile Clustering

Temporal expression patterns were resolved by fuzzy c-means clustering with the R package Mfuzz (v.2.62.0). Variance-stabilizing-transformed (VST) expression values from DESeq2, averaged across biological triplicates per stage, served as input. The cluster number was set to nine, and the fuzzifier coefficient m was estimated from the data with the mestimate function. Functional enrichment was then performed on the gene set assigned to each cluster.

### 2.8. lncRNA–mRNA Co-Expression Network Construction

A signed weighted gene co-expression network was built from the combined DEG–DEL expression matrix with the WGCNA R package (v.1.74) [23]. The soft-thresholding power was selected on the basis of the scale-free topology criterion. Co-expressed transcripts were grouped into modules by average-linkage hierarchical clustering (merge cut height = 0.25). The soft-thresholding power (β) was selected as the lowest integer at which the scale-free topology fit index (R^2^) exceeded 0.85, following the standard WGCNA protocol. Module eigengenes were correlated with developmental stage, and hub genes were defined by module membership (kME > 0.8) and gene significance (GS > 0.4).

### 2.9. Validation of Gene Expression by qPCR

Selected lncRNAs, their co-expressed mRNA partners, and key pathway-related genes were validated by qPCR. Primers (Table S1) were designed with Primer 3.0 and checked by NCBI Primer-BLAST (https://www.ncbi.nlm.nih.gov/tools/primer-blast/; accessed on 15 November 2025). cDNA was synthesized from total RNA with PrimeScript RT Master Mix (Takara, Dalian, China). Reactions were run in triplicate (three biological replicates per stage, each with three technical replicates) using TB Green Premix Ex Taq II (TaKaRa, Dalian, China) on a CFX96 Real-Time PCR Detection System (Bio-Rad, Richmond, CA, USA). Relative expression was calculated by the 2^−ΔΔCq^ method with GAPDH as the internal reference. For each gene, expression values were independently normalized to the maximum observed across the four stages (max = 1.0), as is standard for multi-gene qPCR panels in co-expression studies. This normalization preserves relative trends for cross-stage comparison, while absolute expression differences between lncRNAs and mRNAs are shown in the accompanying RNA-seq panels as log_2_(TPM + 1).

## 3. Results

### 3.1. Plasma Hormone Concentrations and Histological Changes

**Figure 1:**
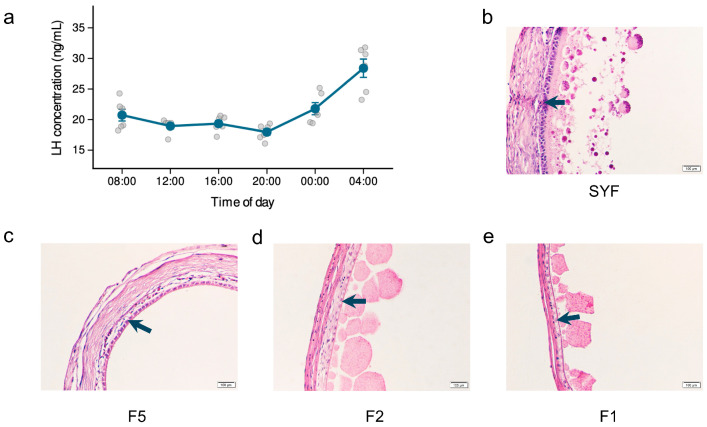
LH profile and histological features of ovarian follicles at the preovulatory surge. (**a**) Serum LH concentrations measured by ELISA at 4 h intervals across a 24 h cycle (*n* = 6 per time point). (**b**–**e**) Representative H&E sections of SYF, F5, F2, and F1 follicles. Arrows indicate granulosa cell layers. Scale bars, 100 μm.

Serum LH concentrations measured by ELISA at six time points across a 24 h cycle were stable during the light phase (~19–20 ng/mL) at 08:00, 12:00, and 16:00; declined to approximately 18 ng/mL at 20:00; rose at 00:00; and reached the observed peak at 04:00 (approximately 28 ng/mL) (Figure 1a). Histological examination of SYF through F1 follicles collected at 04:00 from separate birds in the same experimental cohort showed progressive thinning of the thecal and granulosa layers (Figure 1b–e). SYF contained at least two layers of flattened and cuboidal granulosa cells, F5 retained a single cuboidal layer, and F2 and F1 follicles showed a thin flattened granulosa layer. For H&E staining, the SYF section was prepared from the largest follicle within the 6–8 mm SYF class. Granulosa tissue from these four stages was then collected at the LH surge for RNA extraction.

### 3.2. Data Summary and Genomic Characterization

**Figure 2:**
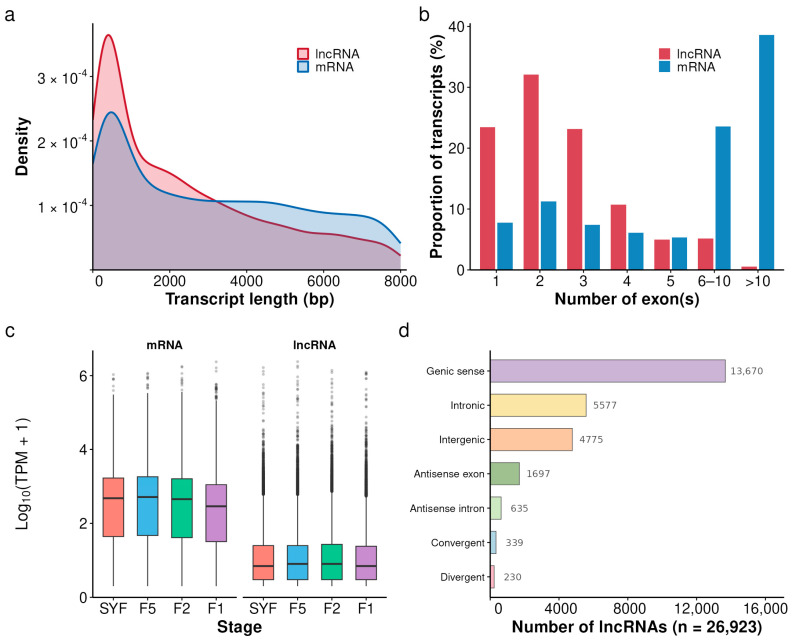
Genomic features of chicken granulosa cell transcripts across developmental stages. (**a**) Transcript length distribution of lncRNAs and mRNAs. (**b**) Distribution of exon numbers in lncRNAs and mRNAs. (**c**) Expression levels of mRNAs and lncRNAs across the four developmental stages; horizontal lines within boxes indicate medians. (**d**) Genomic classification of lncRNAs.

Paired-end sequencing (150 bp) yielded 185.71 Gb of raw data. After adapter trimming and quality filtering, an average of ~15.6 Gb of clean data per sample remained for downstream analysis. Expression filtering (TPM > 0.1 in at least one library) retained 15,838 mRNAs. lncRNAs were annotated through a stringent pipeline requiring concordant non-coding classification by CPC2, CNCI, and CPAT, together with PfamScan screening to remove transcripts harboring known protein domains; 26,923 lncRNAs passed all filters. Compared with mRNAs, lncRNAs were shorter, contained fewer exons, and showed lower expression levels (Figure 2a–c). On the basis of genomic context, FEELnc classified the lncRNAs into seven categories: 13,670 genic sense, 5577 intronic, 4775 intergenic, 1697 antisense exon, 635 antisense intron, 339 convergent, and 230 divergent (Figure 2d).

### 3.3. Expression Patterns of mRNAs and lncRNAs

**Figure 3:**
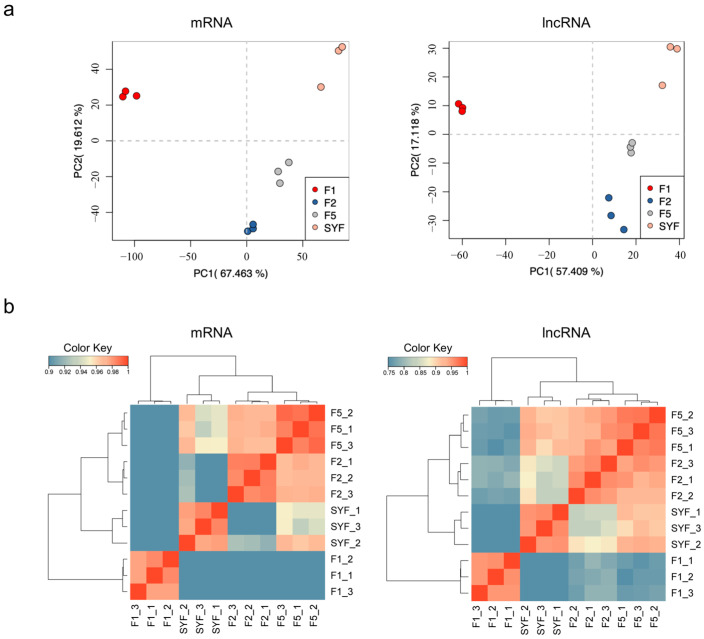
Expression profiles of granulosa cells across follicle stages at the LH surge. (**a**) PCA plots for mRNA (**left**) and lncRNA (**right**) expression. (**b**) Pairwise Pearson correlation heatmaps of mRNA (**left**) and lncRNA (**right**) expression profiles. Dendrograms indicate hierarchical sample clustering; the color scale represents Pearson correlation coefficients.

Separate PCA of mRNA and lncRNA profiles was used to assess stage separation and replicate consistency. In the mRNA dataset, the 12 granulosa cell libraries resolved into four stage-specific clusters (PC1 = 67.5%, PC2 = 19.6%; combined 87.1% of total variance; Figure 3a, left). An independent PCA of lncRNA expression showed the same stage ordering (PC1 = 57.4%, PC2 = 17.1%; Figure 3a, right), with a lower proportion of variance explained by PC1. SYF and F1 were most strongly separated along PC1 in both analyses, whereas F5 and F2 clustered close together. Pairwise Pearson correlation heatmaps also showed high reproducibility among biological replicates and stronger transcriptomic similarity between F5 and F2 than between either stage and SYF or F1 (Figure 3b).

### 3.4. Functional Enrichment Analysis of DEGs and DELs

**Figure 4:**
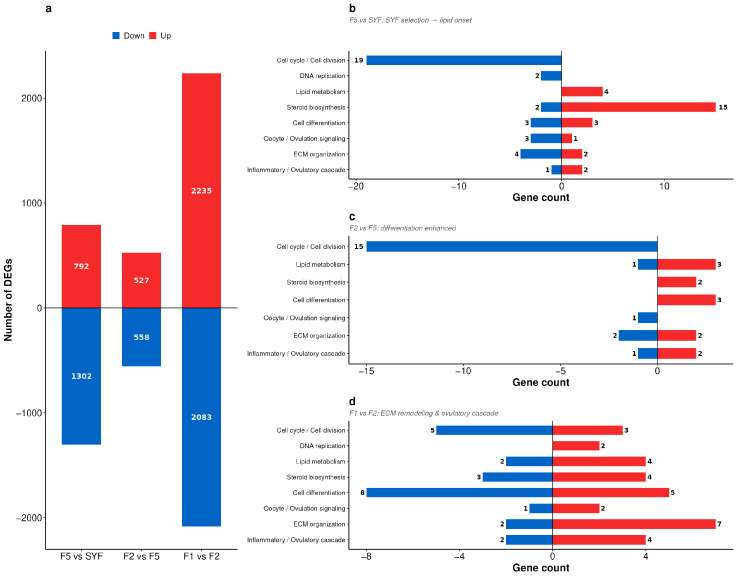
GO and pathway enrichment of DEGs during sequential follicle development. (**a**) Numbers of DEGs in sequential pairwise comparisons (F5 vs. SYF, F2 vs. F5, and F1 vs. F2). (**b–d**) Enriched GO terms and KEGG pathways for each comparison. Up-regulated genes are shown in red and down-regulated genes in blue.

Differential expression analysis across three consecutive pairwise comparisons (F5 vs. SYF, F2 vs. F5, and F1 vs. F2) yielded 2094, 1085, and 4318 DEGs, respectively (Figure 4a, Table S2). The numbers of up- and down-regulated genes were 792/1302 (F5 vs. SYF), 527/558 (F2 vs. F5), and 2235/2083 (F1 vs. F2). At the F1 vs. F2 transition, 3074 of the 4318 DEGs were unique to this step, and the number of DELs increased from 267 in F2 vs. F5 to 2762 in F1 vs. F2, indicating that the F2-to-F1 step accounted for the largest coding and non-coding shift in the sampled hierarchy.

GO and KEGG enrichment showed distinct functional profiles for each comparison. Up-regulated DEGs in F5 vs. SYF were enriched in cholesterol biosynthetic processes, steroid biosynthesis, and lipid metabolism, whereas down-regulated DEGs mapped to cell cycle, DNA replication, and chromosome segregation (Figure 4b, Table S3). In F2 vs. F5, collagen-containing extracellular matrix and cholesterol transfer activity were enriched among up-regulated genes, while cell cycle, kinetochore assembly, and chromosome segregation predominated among down-regulated genes (Figure 4c, Table S3). In F1 vs. F2, up-regulated DEGs were enriched in extracellular-matrix organization, MAPK signaling pathway, and calcium ion binding, whereas down-regulated DEGs were enriched in RET signaling and phosphatidylinositol signaling (Figure 4d, Table S3).

Alongside the coding-gene analysis, 671, 267, and 2762 DELs were detected in F5 vs. SYF, F2 vs. F5, and F1 vs. F2, respectively (Table S4). The numbers of up- and down-regulated DELs were 298/373, 142/125, and 1458/1304, respectively. Putative cis- and trans-target genes were identified using established co-expression criteria (cis: DEGs within 100 kb, |r| > 0.95, *p* < 0.05; trans: genome-wide Pearson correlation, |r| ≥ 0.9, FDR < 0.05). Enrichment analysis of DEL cis-targets showed lipid-metabolism-related terms in F5 vs. SYF, cell-differentiation pathways in F2 vs. F5, and ECM remodeling together with MAPK signaling in F1 vs. F2 (Table S5; Figure S1). Several DELs mapped to the same functional categories across comparisons: notably, *G5825* was linked to lipid-metabolism target genes and *G60212* to DNA-repair-associated targets, and both also appeared as hub lncRNAs in the WGCNA analysis. Stage-specific and shared DEL targets are shown in Figure S2, functional comparison between cis- and trans-regulatory targets in Figure S3, and cis/trans-target distribution in Figure S4. KOBAS enrichment of DEL targets for each comparison is provided in Table S6 and Figure S6. DEL target network visualization and hub lncRNA networks appear in Figures S7 and S8, respectively. The chromosomal distribution of DEL cis-targets is shown in Figure S9, the up- vs. down-regulated DEL functional comparison in Figure S10, and the DEL KOBAS enrichment dotplot in Figure S11.

To address the hierarchy-spanning relationship between SYF and F1, the direct F1 vs. SYF contrast yielded 5899 DEGs (2983 up-regulated in F1 and 2916 down-regulated) and 3675 DELs (1857 up and 1818 down; Table S11). Of the F1 vs. SYF DEGs, 4387 (74.4%) were shared with at least one consecutive comparison. Genes up-regulated in F1 were enriched in extracellular-matrix organization and MAPK signaling, whereas genes down-regulated in F1 mapped mainly to cell cycle, steroid biosynthesis, and lipid metabolism (Figure S12).

### 3.5. Time-Series Clustering of DEGs and DELs

**Figure 5:**
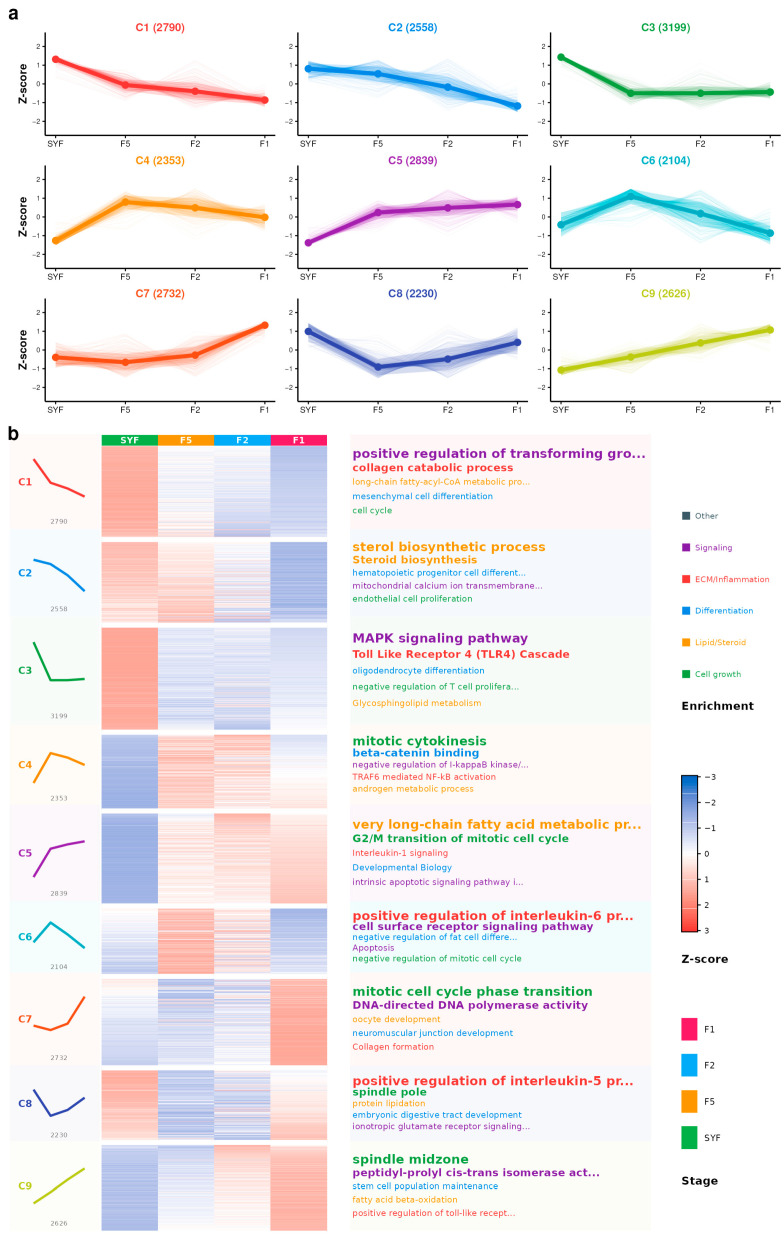
Fuzzy c-means clustering (Mfuzz) of differentially expressed genes and lncRNAs during follicle development. (**a**) Nine temporal expression clusters; each line represents one transcript. Clusters are grouped by peak stage: SYF-peak (Clusters 1–3 and 8), F5-peak (Clusters 4 and 6), and F1-peak (Clusters 5, 7, and 9). (**b**) Gene expression heatmap and representative enrichment terms for each cluster, colored by functional category.

Mfuzz clustering was applied to the combined DEG–DEL matrix to summarize temporal patterns across the four stages. Nine distinct expression patterns were identified (fuzzifier m estimated by the mestimate function; Figure 5, Table S7). Four SYF-peak clusters (Clusters 1–3 and 8; *n* = 10,777) were enriched in TGF-β signaling, sterol biosynthesis, MAPK signaling, and cell division. Two F5-peak clusters (Clusters 4 and 6; *n* = 4457) were associated with NF-κB signaling and endocytosis. Three F1-peak clusters (Clusters 5, 7, and 9; *n* = 8197) were enriched in translation initiation, DNA repair, and cell aging (Table S8; Figure S5). Representative DEGs included *AURKA*, *CDK1*, *CENPE*, and *PLK1* in SYF-peak clusters; *DGAT2*, *SREBF2*, and *ESR1* in F5-peak clusters; and multiple DNA-repair genes in F1-peak clusters. DELs were distributed across all nine clusters (2338 in SYF-peak, 1206 in F5-peak, and 1766 in F1-peak clusters). The highest-membership lncRNAs were *G30477* (Cluster 3, membership = 0.86) and *G37116* (Cluster 8) in SYF-peak clusters, *G677* (Cluster 4) in F5-peak clusters, and *G40971* (Cluster 5, membership = 0.82) and *G42593* (Cluster 7) in F1-peak clusters.

### 3.6. Integrative Co-Expression Analysis of lncRNAs and mRNAs

**Figure 6:**
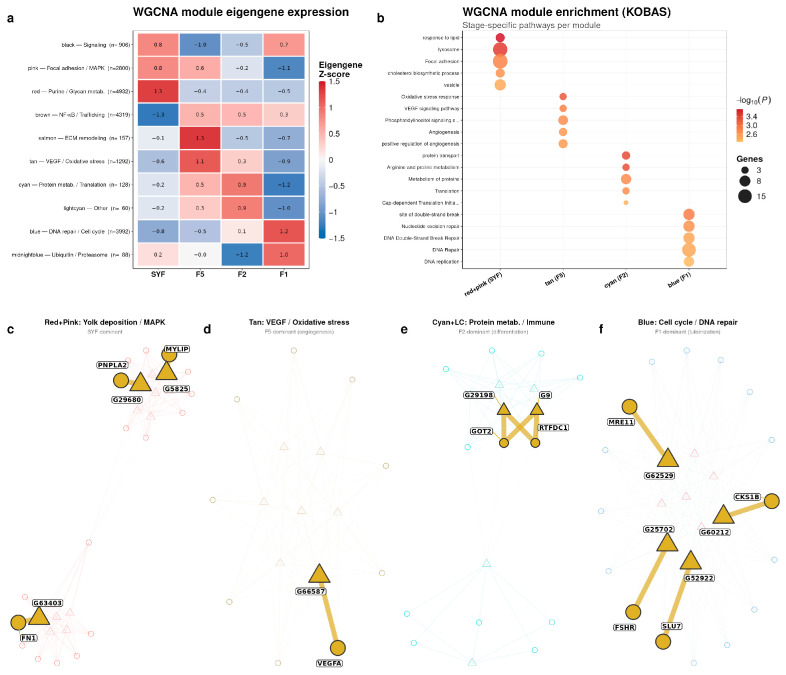
WGCNA of differentially expressed transcripts during follicle development. (**a**) Module eigengene heatmap showing stage-specific expression patterns across 10 co-expression modules. (**b**) KOBAS enrichment bubble plot for representative modules. (**c**) Merged red and pink co-expression network (SYF-dominant). (**d**) Tan module co-expression network (F5-dominant). (**e**) Merged cyan and lightcyan co-expression network (F2-dominant). (**f**) Blue module co-expression network (F1-dominant). Triangles indicate lncRNAs, and circles indicate mRNAs. Gold nodes with boxed labels denote qPCR-validated lncRNA-mRNA pairs.

To determine whether lncRNAs and protein-coding genes form coordinated stage-specific modules, a signed WGCNA network was constructed from the combined DEG–DEL expression matrix. Ten co-expression modules were identified whose eigengene profiles displayed clear stage-specific patterns (Figure 6a, Table S9). The red (*n* = 4932), pink (*n* = 2800), and blue (*n* = 3992) modules were the three largest. KOBAS pathway enrichment assigned lysosome, purine metabolism, and N-glycan biosynthesis to the SYF-dominant red module; lipid response, focal adhesion, cholesterol biosynthetic process, and MAPK signaling to the pink module; VEGF signaling, oxidative stress response, and angiogenesis to the F5-dominant tan module; and nucleotide excision repair, DNA repair, DNA replication, and cell cycle to the F1-dominant blue module (Figure 6b, Table S10). Hub genes (kME > 0.8, GS > 0.4) were visualized in co-expression networks for four stage-representative module groups (Figure 6c–f); qPCR-validated lncRNA–mRNA pairs are marked by gold-colored nodes.

The SYF-dominant red and pink modules were merged for network visualization (Figure 6c). Hub lncRNAs *G29680* and *G5825* in the red module were co-expressed with PNPLA2 and MYLIP, respectively. In the pink module, hub mRNAs *LPL* and *VTG2* appeared alongside hub lncRNA *G63403*, which was co-expressed with *FN1*. The F5-dominant tan module (Figure 6d) contained hub lncRNA *G66587* co-expressed with *VEGFA*.

The cyan and lightcyan modules (Figure 6e) showed F2-dominant eigengene values and were enriched in protein metabolism, translation, and immune-related pathways. The F1-dominant blue module (Figure 6f) was enriched in DNA repair and cell cycle pathways; hub lncRNAs *G60212*, *G62529*, *G52922*, and *G25702* were co-expressed with *CKS1B*, *MRE11*, *SLU7*, and *FSHR*, respectively.

Cross-tabulation of Mfuzz clusters and WGCNA modules showed that SYF-peak clusters (1–3 and 8) overlapped mainly with the SYF-dominant red and pink modules, including 778 of 870 Cluster-1 members in the red module and 831 of 881 Cluster-2 members in the pink module. F1-peak clusters (5, 7, and 9) converged on the F1-dominant blue module, whereas F5-peak clusters (4 and 6) aligned with the tan module and an additional brown module in the cross-tabulation (Figure S13), linking temporal expression classes with stage-associated co-expression modules.

### 3.7. Validation of Key Genes and lncRNA–mRNA Pairs by qPCR

qPCR was performed for five functional marker genes and eight lncRNA–mRNA pairs to verify RNA-seq expression profiles and the co-expression relationships identified by WGCNA. For all tested transcripts, qPCR trends were concordant with the sequencing data across the four follicle stages.

**Figure 7:**
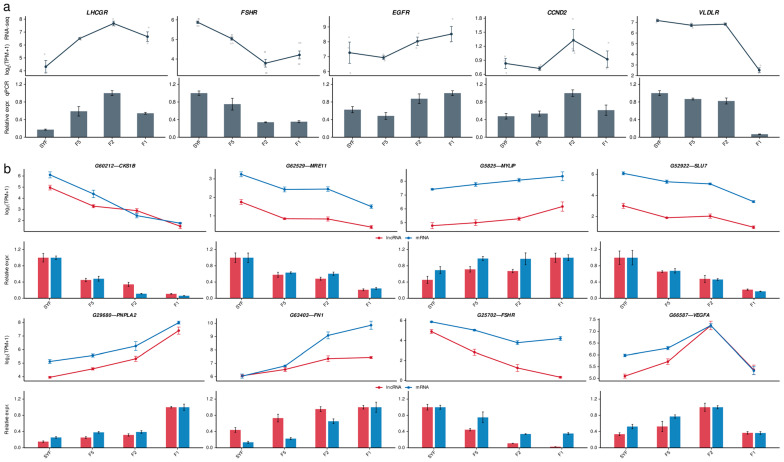
qPCR validation of key genes and co-expressed lncRNA–mRNA pairs during follicle development. (**a**) RNA-seq and qPCR expression profiles of five representative genes (*LHCGR*, *FSHR*, *EGFR*, *CCND2*, and *VLDLR*) across follicle stages. (**b**) RNA-seq and qPCR expression profiles of eight validated lncRNA–mRNA pairs: *G60212*–*CKS1B*, *G62529*–*MRE11*, *G5825*–*MYLIP*, *G52922*–*SLU7*, *G29680*–*PNPLA2*, *G63403*–*FN1*, *G25702*–*FSHR*, and *G66587*–*VEGFA*. qPCR values were normalized to *GAPDH* and scaled to each gene’s maximum across stages. Error bars represent SEM of three biological replicates.

The five functional marker genes selected for qPCR, *LHCGR, FSHR, EGFR, CCND2*, and *VLDLR*, were used to anchor the expression profiles to established granulosa cell programs rather than to represent candidate lncRNA targets. They mark gonadotropin responsiveness (*LHCGR*, *FSHR*), EGF-like signaling (*EGFR*), proliferative capacity (*CCND2*), and yolk-lipid uptake (*VLDLR*) in avian follicles. *LHCGR* expression increased from SYF to F2 and declined at F1, whereas *EGFR* showed its highest expression at F1 (Figure 7a). *FSHR* decreased from SYF through F2 and then increased at F1. *CCND2* showed its highest expression at F2 and lower expression at F1, and *VLDLR* reached its lowest level at F1 (Figure 7a).

Eight lncRNA–mRNA pairs were chosen for validation based on hub status (kME > 0.8), co-expression strength (|r| > 0.9), and pathway annotation. The validated lncRNAs ranged from 487 to 72,101 nt and were classified by FEELnc as intergenic (*n* = 4), genic sense (*n* = 3), or antisense (*n* = 1) (Table 1). *G5825*–*MYLIP* and *G29680*–*PNPLA2* met the putative cis criteria, five pairs met the genome-wide trans co-expression criteria, and *G52922*–*SLU7* was retained as a WGCNA-supported co-expression pair. Together, these data provide genomic context and putative regulatory classifications for the validated lncRNA–mRNA candidates.

All eight pairs showed expression profiles consistent with the RNA-seq data (Figure 7b). Within the blue module (F1-dominant), *G60212*–*CKS1B*, *G62529*–*MRE11*, *G52922*–*SLU7*, and *G25702*–*FSHR* showed parallel stage-dependent expression changes. In the red module (SYF-dominant), *G29680*–*PNPLA2* and *G5825*–*MYLIP* also showed parallel changes across stages. The pink module pair *G63403*–*FN1* showed parallel stage-dependent expression, and the tan module pair *G66587*–*VEGFA* showed higher expression in early hierarchical follicles (F5/F2), consistent with the tan module pattern.

## 4. Discussion

Collecting all four follicle classes at the preovulatory LH surge allowed stage-dependent differences to be interpreted on a common endocrine background rather than across disparate points of the ovulatory cycle—a design distinction that sharpens attribution of transcriptomic shifts to maturational state rather than hormonal fluctuation. Prior chicken studies typically juxtaposed prehierarchical and preovulatory fractions or examined single candidate regulators. Du et al. [6] documented broad transcriptomic differences between prehierarchical and preovulatory granulosa cells, noting stronger steroid-biosynthetic signatures and progressive layer thinning in the larger follicles. Zhong et al. [4] showed that FSH reshapes the transcriptional program in prehierarchical cells, while Shen et al. [7] subsequently identified *SLC5A5* as a regulator of proliferation, apoptosis, and steroid synthesis in the same compartment. The present four-stage dataset extends this resolution across the full hierarchical series and places the most extensive transcriptional reorganization at the F2-to-F1 transition.

At the earlier stages, SYF and newly recruited hierarchical follicles in our dataset were marked by cell-cycle activity, lipid handling, and steroidogenic preparation—consistent with the rapid growth and granulosa cell differentiation that follow escape from inhibitory selection pressure [2]. This interpretation is supported by functional studies in chicken granulosa cells: Gong et al. [24] showed that Noggin4 modulates cell proliferation and differentiation, and Deng et al. [25] reported that miR-22-3p drives proliferation, steroidogenesis, and lipid metabolism through PTEN/PI3K/Akt/mTOR signaling in hierarchical follicles. The tight clustering of F5 and F2 in our PCA supports gradual developmental continuity rather than a discrete transition at that point, with cholesterol metabolism, vascular support, and endocrine competence reinforced progressively as follicles advance. *DHCR7*, chemerin, and the miR-15c-3p/*IGF2BP3* axis have each been reported to modulate cholesterol handling, progesterone secretion, and lipid storage in granulosa cells under oxidative conditions [26,27,28]. Evidence from bovine and human granulosa cell models likewise links metabolic remodeling to steroidogenic output, pointing to a conserved coupling between energy status and hormone synthesis [29,30], and a parallel relationship between ERK1/2 activity, steroidogenesis, and cell fate has been documented in sheep granulosa cells exposed to kisspeptin [31].

The largest transcriptional shift in our dataset, occurring between F2 and F1, was centered on extracellular-matrix organization and MAPK signaling—hallmarks of the periovulatory remodeling state. Matrix metalloproteinases and their inhibitors are known to fluctuate during chicken follicle development and atresia [9], and follicle rupture has been shown to require coordinated proteolytic, vascular, and inflammatory activity [32]. ERK1/2 activity is itself required for ovulatory differentiation of granulosa cells [33]. At the stage level, miR-128-3p is more abundant in F1 than F5 granulosa cells and suppresses lipid synthesis and steroid output while promoting apoptosis [34]—a pattern that, combined with the declining *LHCGR* and *CCND2* signals we observed at F1, argues that the dominant follicle has largely withdrawn from growth-associated transcription and committed to tissue remodeling and damage-pathway reorganization in preparation for rupture. Regulators of oxidative stress, apoptosis, and autophagy have likewise been implicated in granulosa cell dysfunction and follicular failure, underscoring the importance of stress-response competence at this stage [35,36].

The non-coding candidates were prioritized using convergent evidence from differential expression, WGCNA module assignment, cis-target prediction, and qPCR validation; however, their causal roles will require targeted perturbation experiments. Non-coding RNAs are functional in granulosa cells at multiple levels: lncRNA-*FMR6* promotes apoptosis in mammalian models, and *NEAT1* influences both apoptosis and estradiol synthesis in ovarian dysfunction [12,13,37]. In chicken granulosa cells, He et al. [38] reported that circRALGPS2 promotes granulosa cell apoptosis and autophagy, and Han et al. [39] identified USP13 as a regulator of ATG7-dependent ferroptosis. Within this context, the validated pairs *G5825*–*MYLIP*, *G66587*–*VEGFA*, *G63403*–*FN1*, *G62529*–*MRE11*, and *G60212*–*CKS1B* provide biologically interpretable candidates for such follow-up work.

These findings should be interpreted in light of several constraints. Sampling was restricted to the preovulatory LH surge, defined from serum LH profiles based on *n* = 6 birds per time point; larger endocrine-profiling cohorts will be needed to assess inter-individual hormonal variation and the stability of these lncRNA–mRNA relationships across the ovulatory cycle. The study used Lohmann Pink-shell layers only, so breed-specific expression cannot be excluded. In addition, bulk RNA-seq and co-expression analysis cannot resolve granulosa cell heterogeneity or establish causality; single-cell/spatial profiling and perturbation experiments will be needed to test direct regulatory roles.

## 5. Conclusions

Granulosa cells in the chicken follicular hierarchy undergo a stepwise transcriptional transition, with the most extensive coding and non-coding reorganization occurring between F2 and F1. Prehierarchical (SYF) and early hierarchical (F5) follicles are dominated by proliferative, metabolic, and steroidogenic programs, while F1 granulosa cells switch to extracellular-matrix remodeling, MAPK signaling, and stress-response pathways. Among the lncRNA–mRNA pairs identified and validated by qPCR, *G5825–MYLIP* and *G29680–PNPLA2* (SYF-dominant, lipid metabolism), *G66587–VEGFA* (F5-dominant, angiogenesis), and *G60212–CKS1B* and *G62529–MRE11* (F1-dominant, DNA repair and cell cycle) represent priority candidates for functional validation. Together, these modules and validated pairs offer tractable targets for testing how non-coding regulation contributes to follicle maturation, periovulatory remodeling, and egg-production traits in laying hens.

## Figures and Tables

**Table 1:** Putative regulatory relationships and network features of eight qPCR-validated lncRNA–mRNA pairs.

lncRNA	Target mRNA	Putative Relationship	Module	kME	Cis/Trans-Targets	Peak Stage
*G25702*	*FSHR*	trans	blue	0.958	2/801	F1
*G66587*	*VEGFA*	trans	tan	0.924	2/222	F5
*G29680*	*PNPLA2*	cis	red	0.963	4/667	SYF
*G63403*	*FN1*	trans	pink	0.897	18/919	SYF
*G62529*	*MRE11*	trans	blue	0.985	3/550	F1
*G60212*	*CKS1B*	trans	blue	0.985	8/961	F1
*G52922*	*SLU7*	WGCNA co-expression	blue	0.982	0/0	F1
*G5825*	*MYLIP*	cis	red	0.908	1/71	SYF

## Data Availability

The raw sequencing data generated in this study have been deposited in the BioProject database at the National Genomics Data Center (NGDC), China National Center for Bioinformation, under accession number PRJCA060696. The dataset will be released upon publication. Additional data supporting the findings of this study are available from the corresponding author upon reasonable request.

## Supplementary Materials

The following supporting information can be downloaded at https://www.mdpi.com/article/10.3390/ani16091351/s1, Table S1, primer sequences and amplicon information used for qPCR validation; Table S2, differentially expressed genes (DEGs) in three consecutive pairwise comparisons (F5 vs. SYF, F2 vs. F5, and F1 vs. F2); Table S3, GO and KEGG enrichment results for DEGs; Table S4, differentially expressed lncRNAs (DELs) in three consecutive pairwise comparisons; Table S5, cis-target genes of DELs together with co-expression statistics, cis/trans-enrichment results, and ranked candidate lncRNA list; Table S6, KOBAS enrichment results for DELs cis- and trans-target genes in each pairwise comparison; Table S7, transcript membership scores in the nine Mfuzz temporal clusters; Table S8, functional enrichment results for each Mfuzz cluster; Table S9, WGCNA module assignment and module membership scores for all differentially expressed transcripts; Table S10, KOBAS enrichment results for the four representative WGCNA modules (blue, red, pink, and tan); Figure S1, GO and KEGG enrichment of DELs cis-target genes for each pairwise comparison; Figure S2, stage-specific and shared DELs target genes across comparisons; Figure S3, functional comparison of DELs cis- and trans-regulatory targets; Figure S4, distribution of DELs cis- and trans-target gene counts; Figure S5, functional enrichment results for each Mfuzz temporal cluster; Figure S6, KOBAS enrichment bar plots for DELs target genes in each pairwise comparison; Figure S7, co-expression network visualization of DELs targets; Figure S8, hub lncRNA co-expression networks; Figure S9, chromosomal distribution of DELs cis-target genes; Figure S10, functional comparison of up- and down-regulated DELs targets; Figure S11, KOBAS enrichment dotplot summary for DELs target genes; Table S11, differentially expressed genes (Table S11a) and lncRNAs (Table S11b) in the direct F1 vs. SYF comparison; Figure S12, GO/KEGG enrichment bubble plot for F1 vs. SYF DEGs; Figure S13, cross-tabulation heatmap of Mfuzz temporal clusters and WGCNA co-expression modules.
