# Supplementary material for: Stage-Specific lncRNA–mRNA Co-Expression Networks in Chicken Granulosa Cells Across Hierarchical Follicle Development
Source: Animals (Basel). 2026 Apr 28;16(9):1351. doi: 10.3390/ani16091351 (PMC13162649; doi:10.3390/ani16091351)
Supplement: Supplementary file 1 [file animals-16-01351-s001.zip › Figure S2_DEL_stage_specific_shared.pdf]

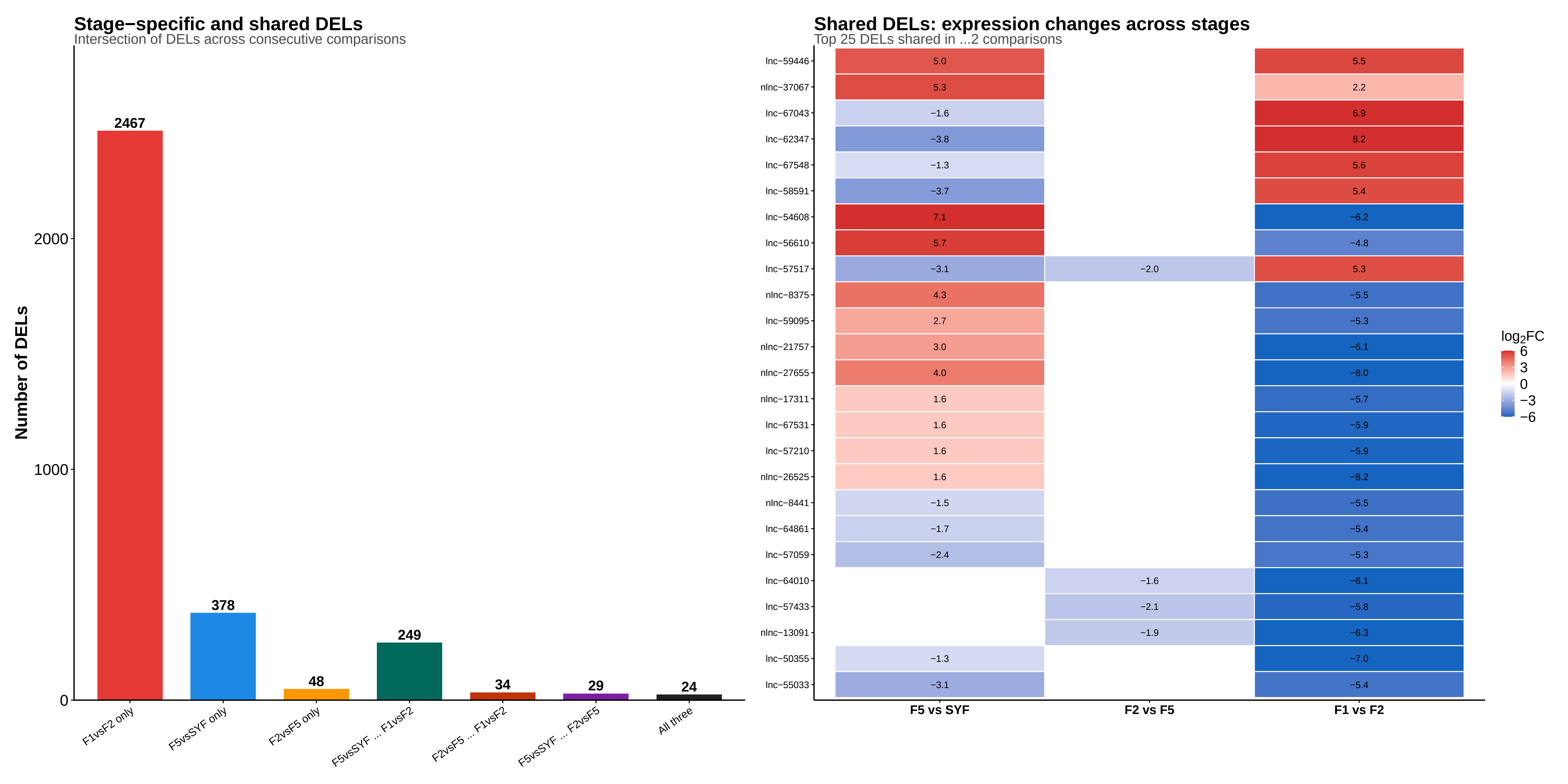
Stage−specific and shared DELs
Intersection of DELs across consecutive comparisons
Number of DELs
0
1000
2000
2467
378
48
249
34
29
24
F1vsF2 only
F5vsSYF only
F2vsF5 only
F5vsSYF ... F1vsF2
F2vsF5 ... F1vsF2
F5vsSYF ... F2vsF5
All three
Shared DELs: expression changes across stages
Top 25 DELs shared in ...2 comparisons
lnc−59446 5.0 5.5
nlnc−37067 5.3 2.2
lnc−67043 −1.6 6.9
lnc−62347 −3.8 8.2
lnc−67548 −1.3 5.6
lnc−58591 −3.7 5.4
lnc−54608 7.1 −6.2
lnc−56610 5.7 −4.8
lnc−57517 −3.1 −2.0 5.3
nlnc−8375 4.3 −5.5
lnc−59095 2.7 −5.3
nlnc−21757 3.0 −6.1
nlnc−27655 4.0 −8.0
nlnc−17311 1.6 −5.7
lnc−67531 1.6 −5.9
lnc−57210 1.6 −5.9
nlnc−26525 1.6 −8.2
nlnc−8441 −1.5 −5.5
lnc−64861 −1.7 −5.4
lnc−57059 −2.4 −5.3
lnc−64010 −1.6 −6.1
lnc−57433 −2.1 −5.8
nlnc−13091 −1.9 −6.3
lnc−50355 −1.3 −7.0
lnc−55033 −3.1 −5.4
F5 vs SYF
F2 vs F5
F1 vs F2
log2FC
6
3
0
−3
−6
