# Supplementary figures and images for: Stage-Specific lncRNA–mRNA Co-Expression Networks in Chicken Granulosa Cells Across Hierarchical Follicle Development
Source: Animals (Basel). 2026 Apr 28;16(9):1351. doi: 10.3390/ani16091351 (PMC13162649; doi:10.3390/ani16091351)

Cis vs Trans target enrichment  
Top pathways enriched in DEL cis/trans target mRNAs

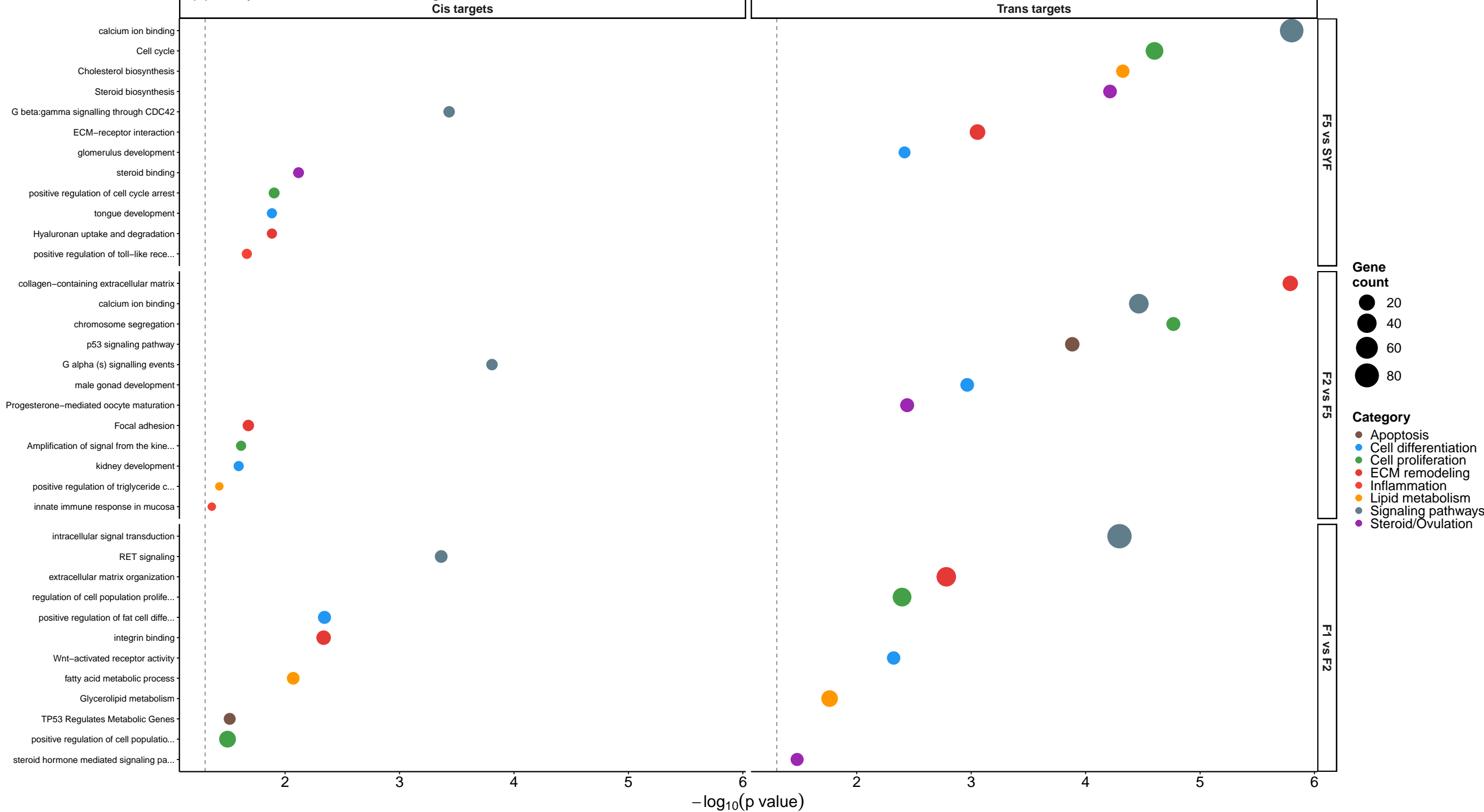

Supplement: Supplementary file 1 [file animals-16-01351-s001.zip › Figure S1_DEL_cis_trans_pathway.pdf]

# Up vs Down DEL target functional comparison

Down Up

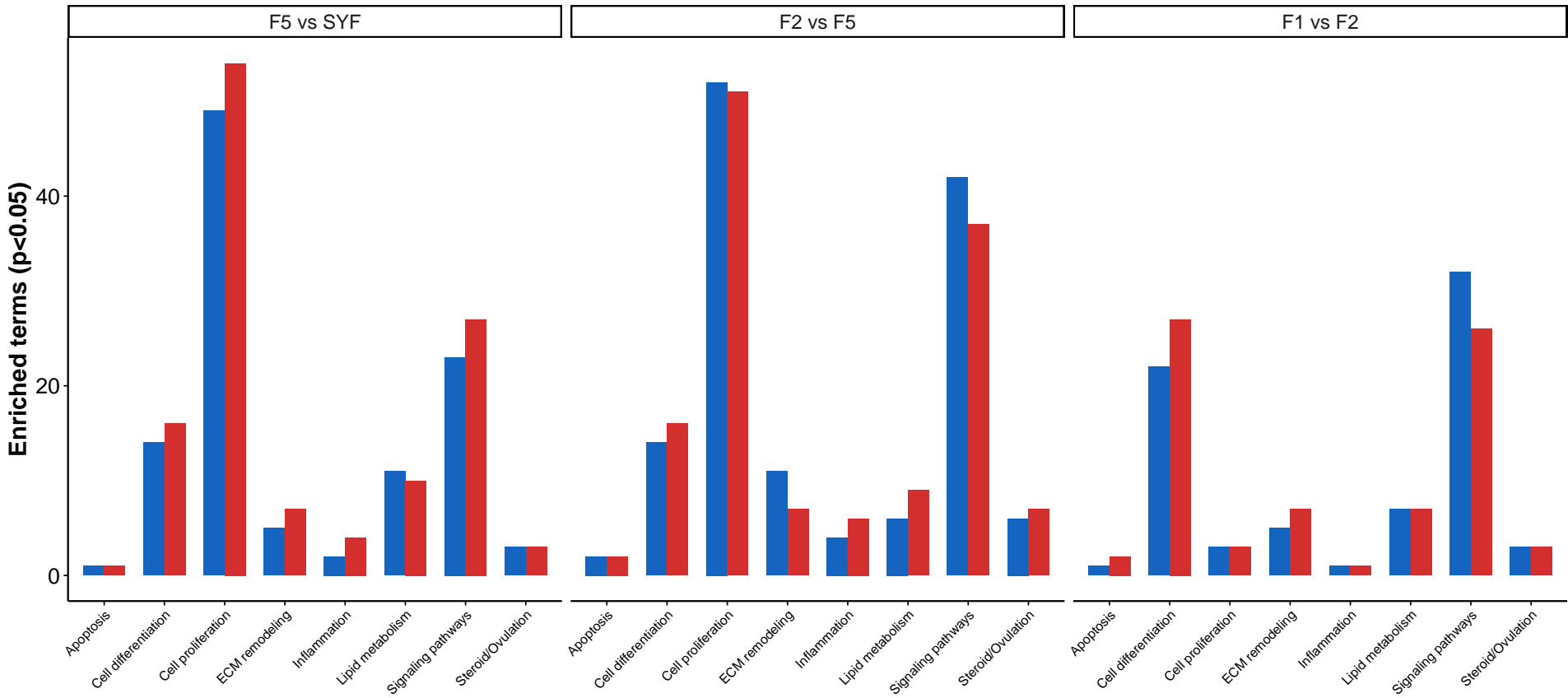

Supplement: Supplementary file 1 [file animals-16-01351-s001.zip › Figure S10_DEL_up_vs_down_function.pdf]

b

## KOBAS enrichment of DEL targets (sequential comparisons)

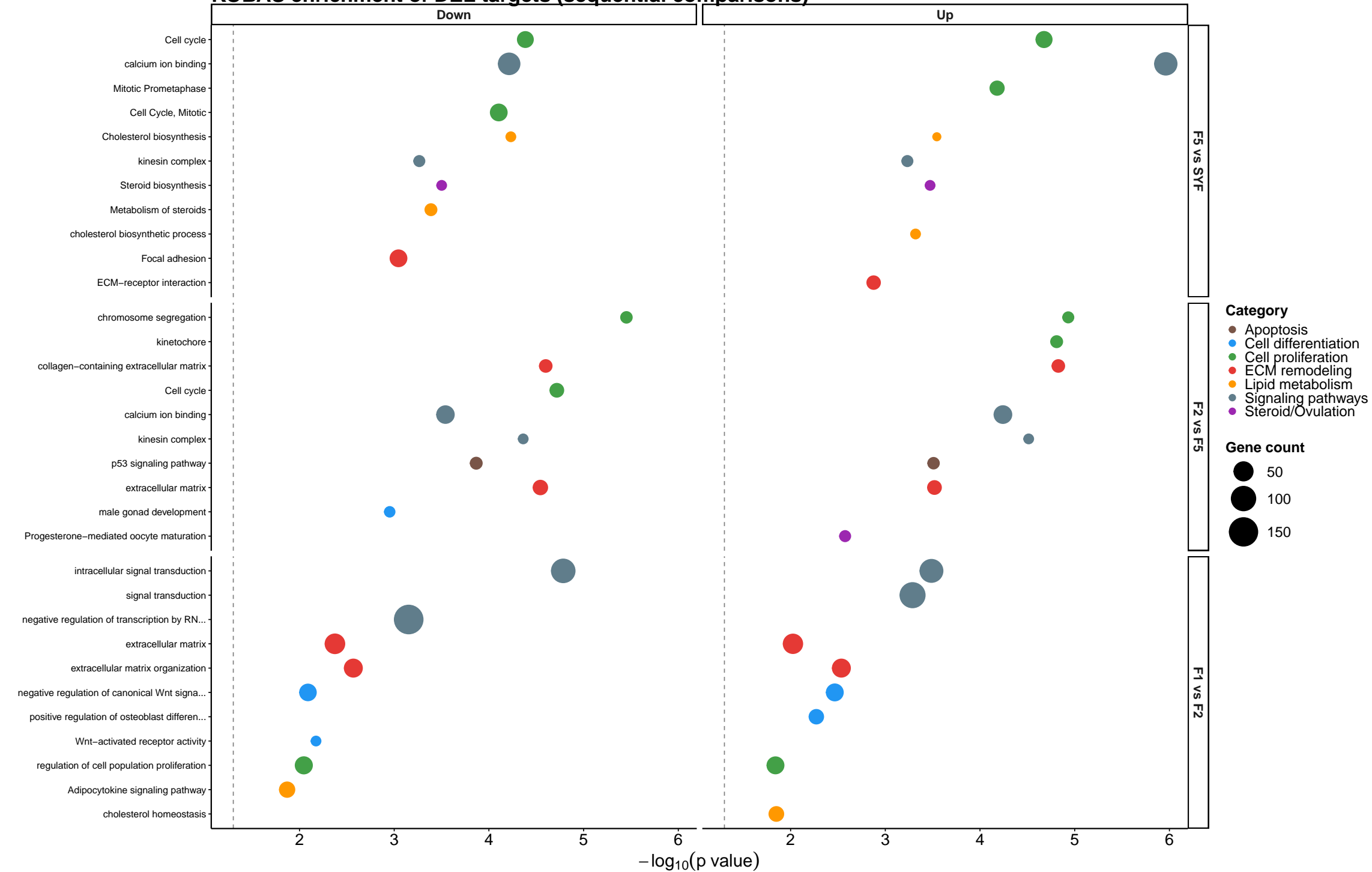

Supplement: Supplementary file 1 [file animals-16-01351-s001.zip › Figure S11_DEL_KOBAS_dotplot.pdf]

# GO/KEGG enrichment: F1 vs. SYF DEGs

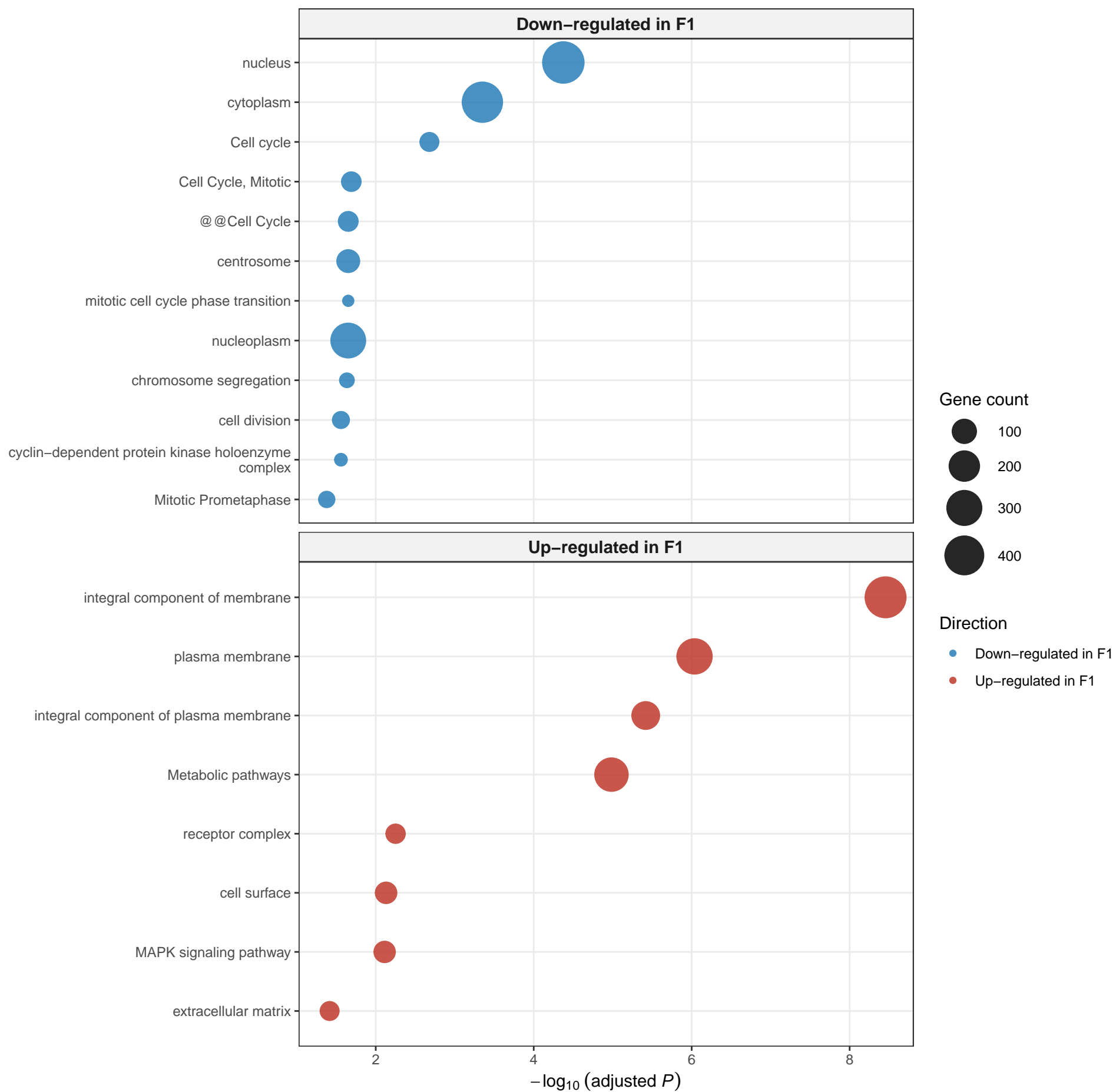

Supplement: Supplementary file 1 [file animals-16-01351-s001.zip › Figure S12_F1vsSYF_enrichment.pdf]

# Concordance between Mfuzz clusters and WGCNA modules

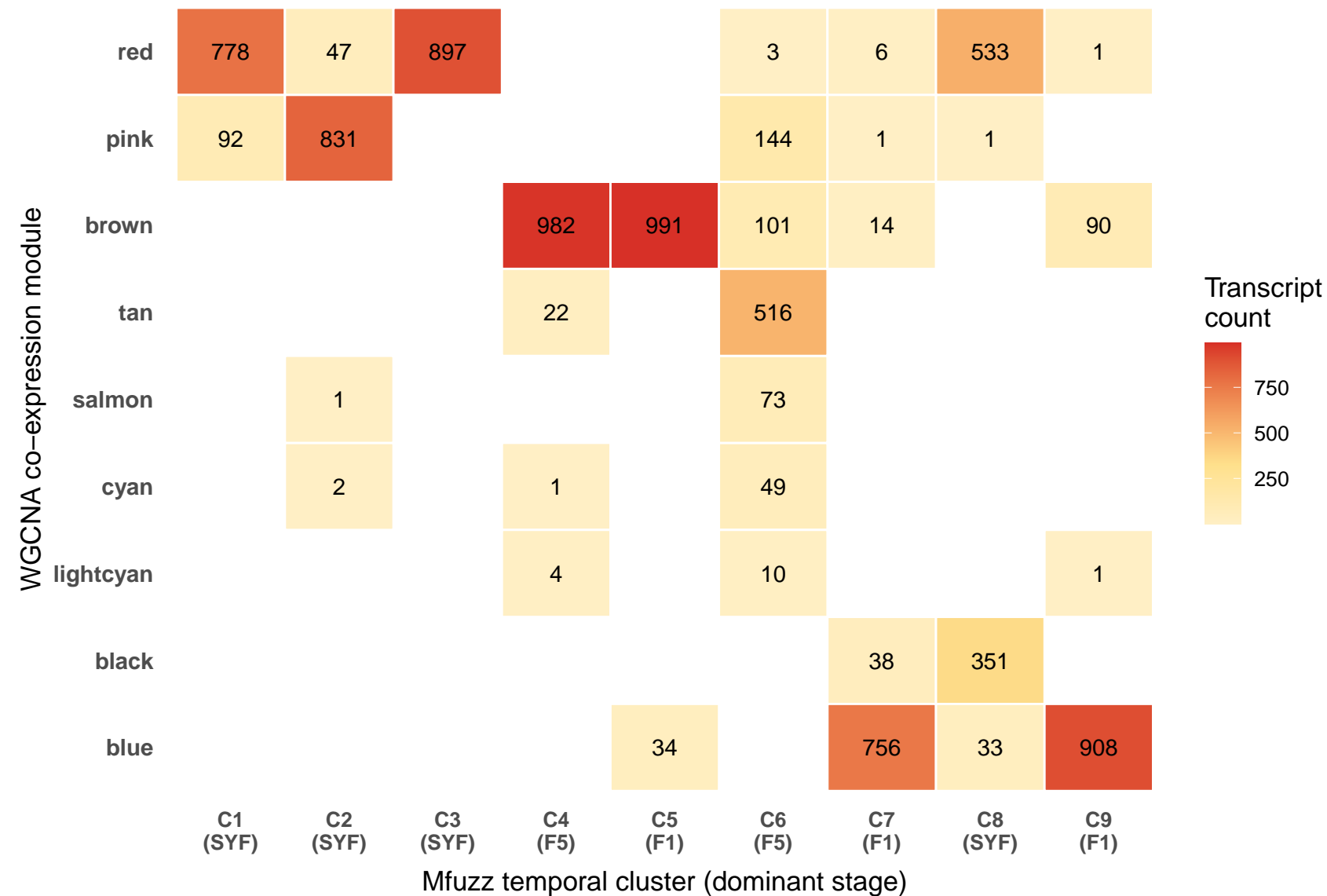

Supplement: Supplementary file 1 [file animals-16-01351-s001.zip › Figure S13_Mfuzz_WGCNA_cross_tabulation.pdf]

# Cis vs Trans: functional enrichment comparison

■ Cis targets ■ Trans targets

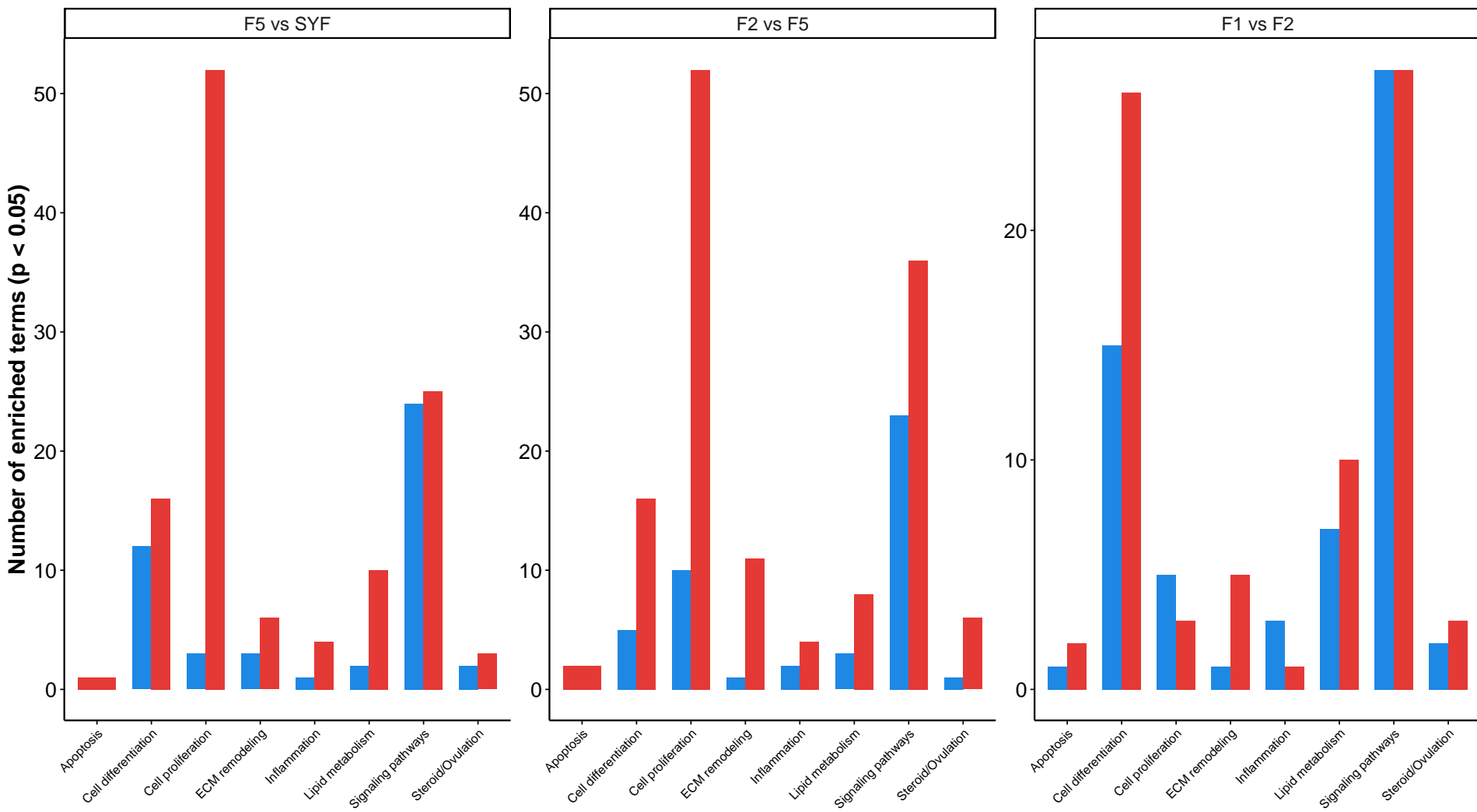

Supplement: Supplementary file 1 [file animals-16-01351-s001.zip › Figure S3_DEL_cis_vs_trans_function.pdf]

### Cis vs Trans distribution

■ Cis ■ Trans

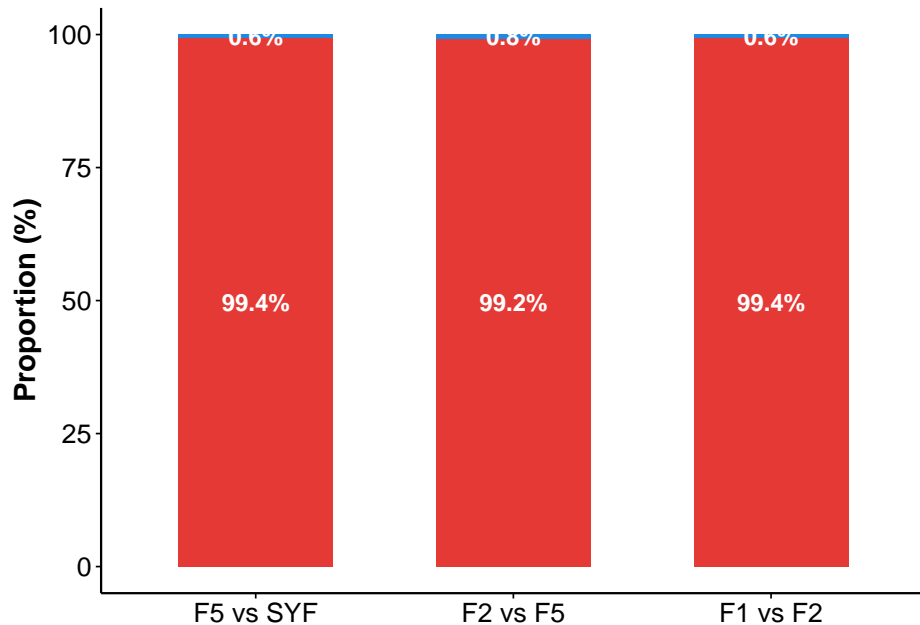

### Target mRNA counts

■ Cis ■ Trans

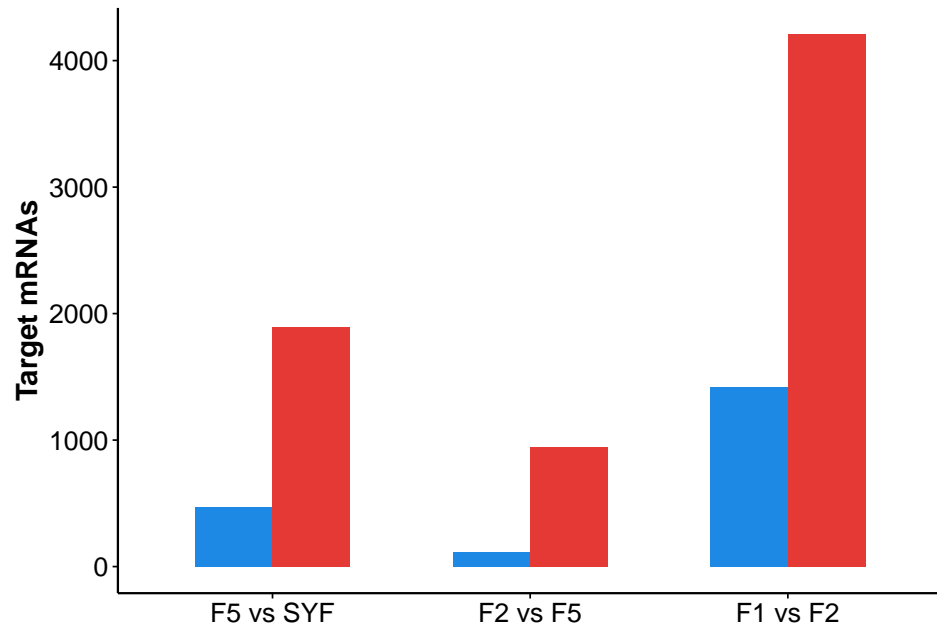

Supplement: Supplementary file 1 [file animals-16-01351-s001.zip › Figure S4_DEL_cis_trans_distribution.pdf]

Cluster 2 (peak SYF, 2,558 genes)

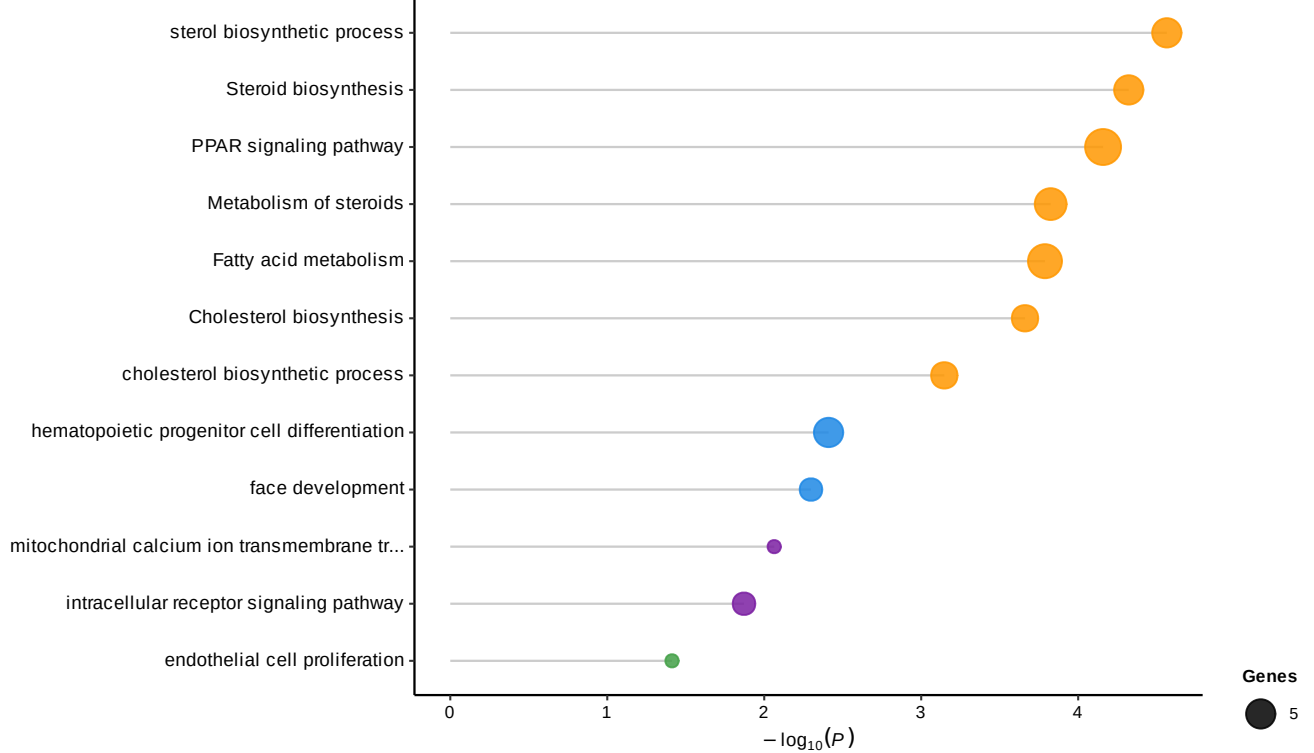

Cluster 7 (peak F1, 2,732 genes)

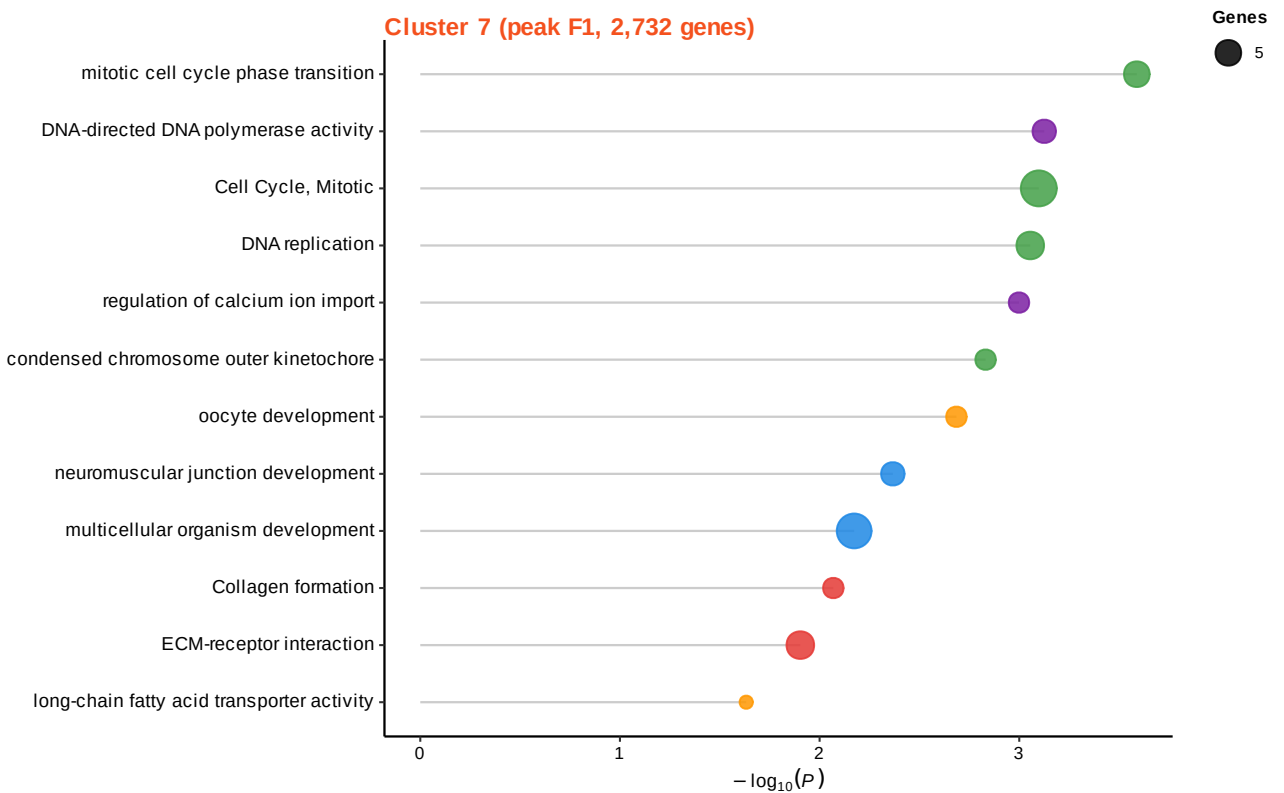

Supplement: Supplementary file 1 [file animals-16-01351-s001.zip › Figure S5_mfuzz_enrichment.pdf]

a

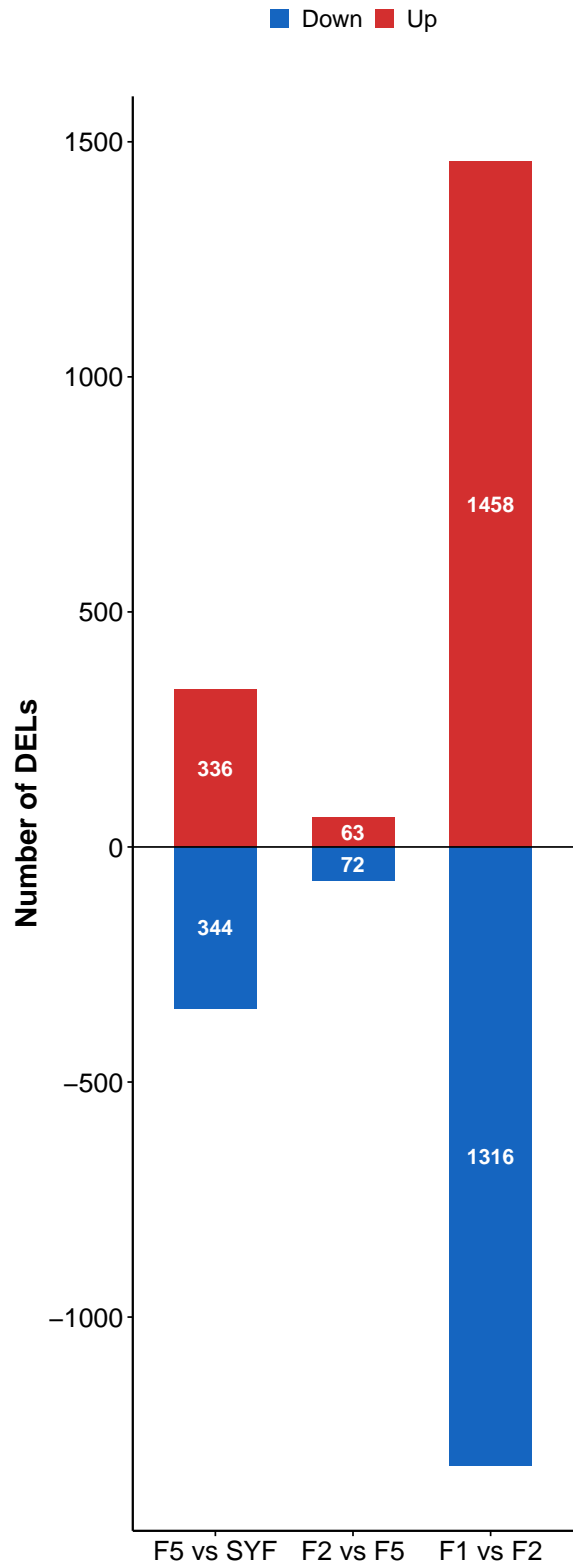

b

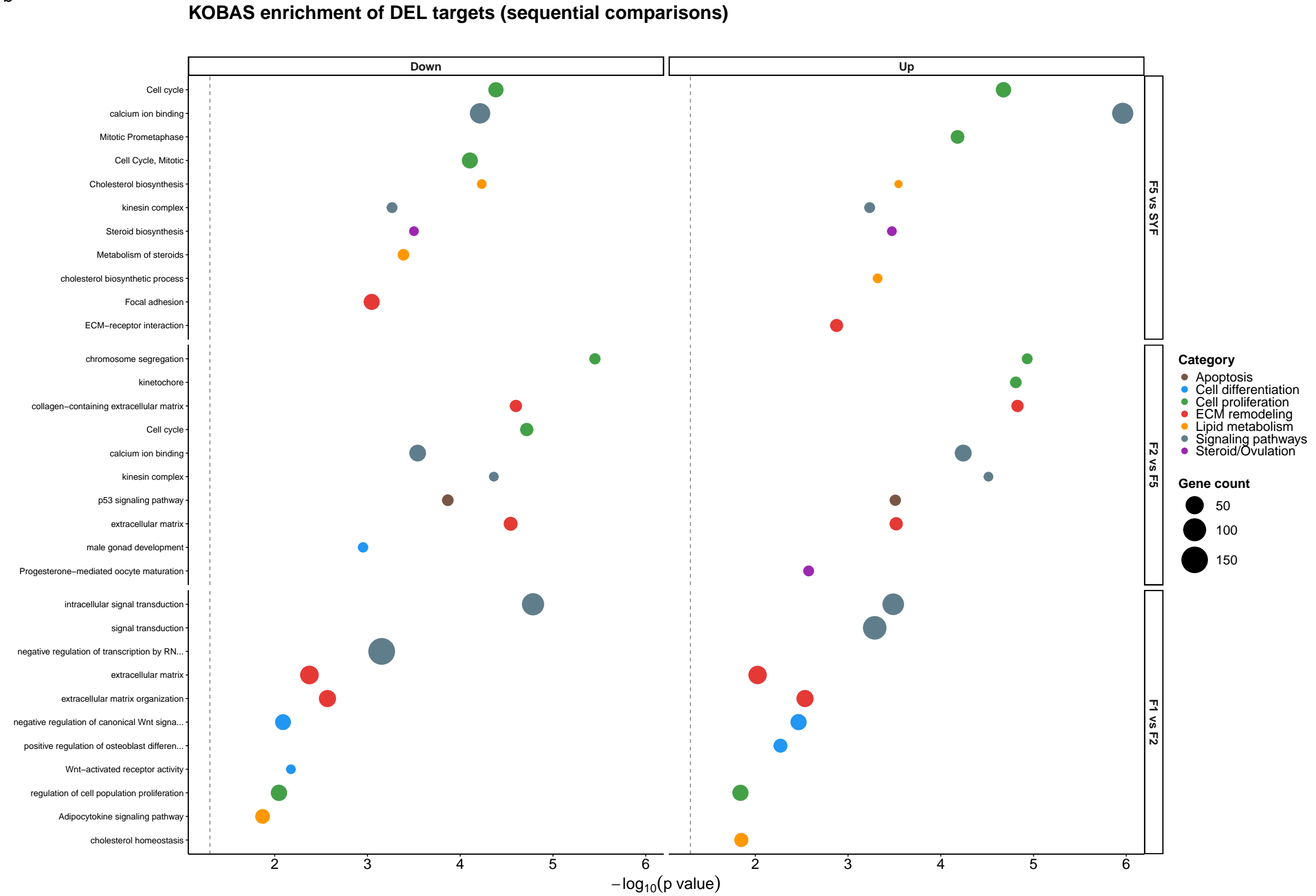

Supplement: Supplementary file 1 [file animals-16-01351-s001.zip › Figure S6_DEL_KOBAS_enrichment.pdf]

### Bio-relevant targets across follicle developmental stages

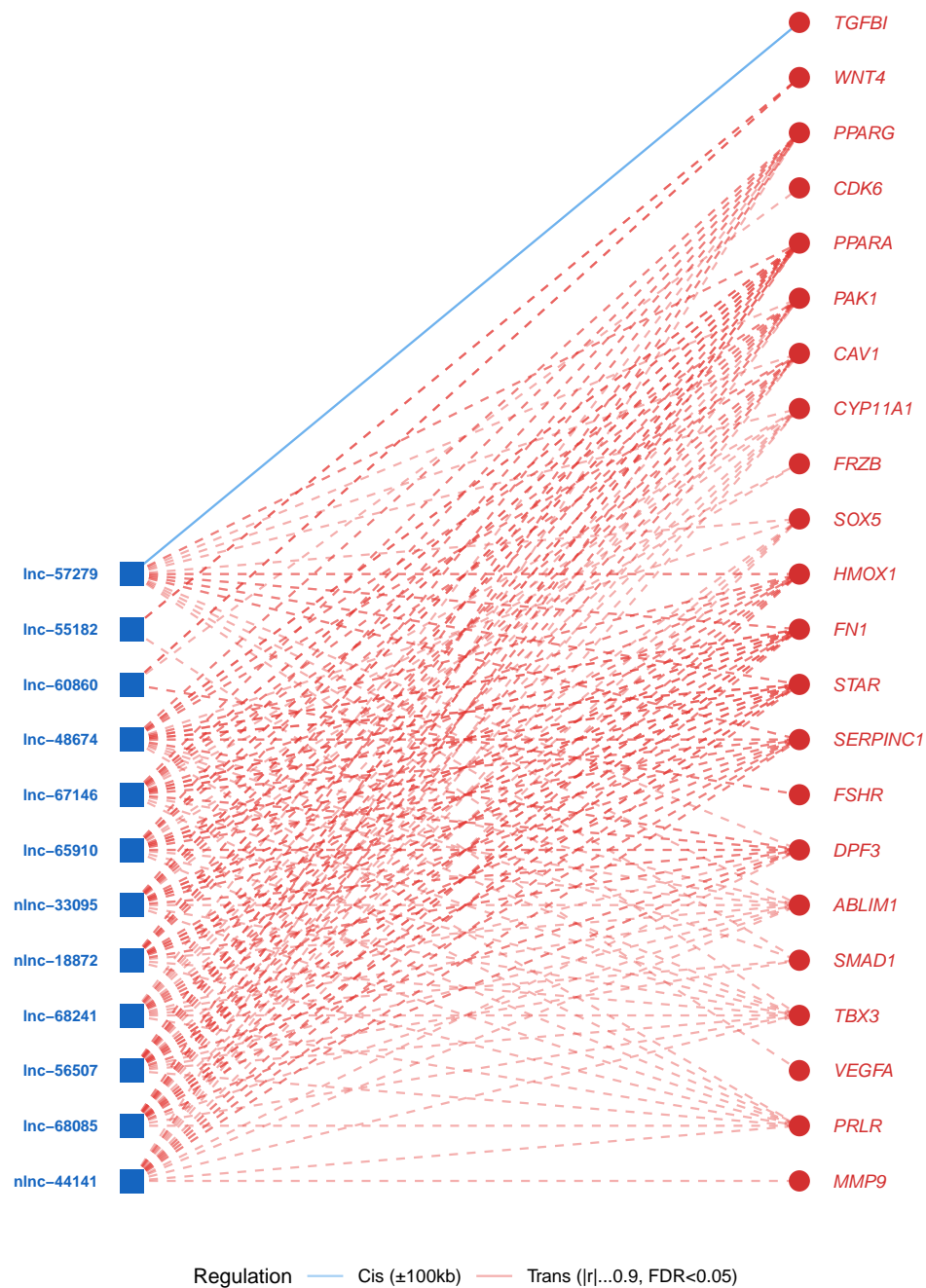

Supplement: Supplementary file 1 [file animals-16-01351-s001.zip › Figure S7_DEL_target_network.pdf]

Top 10 hub DELs

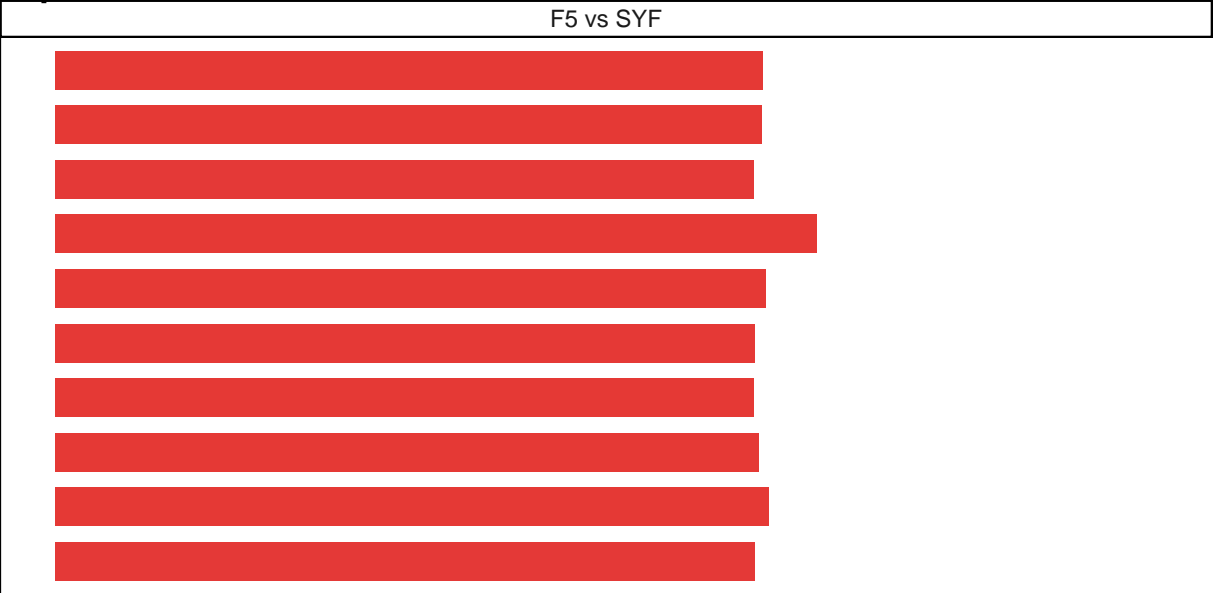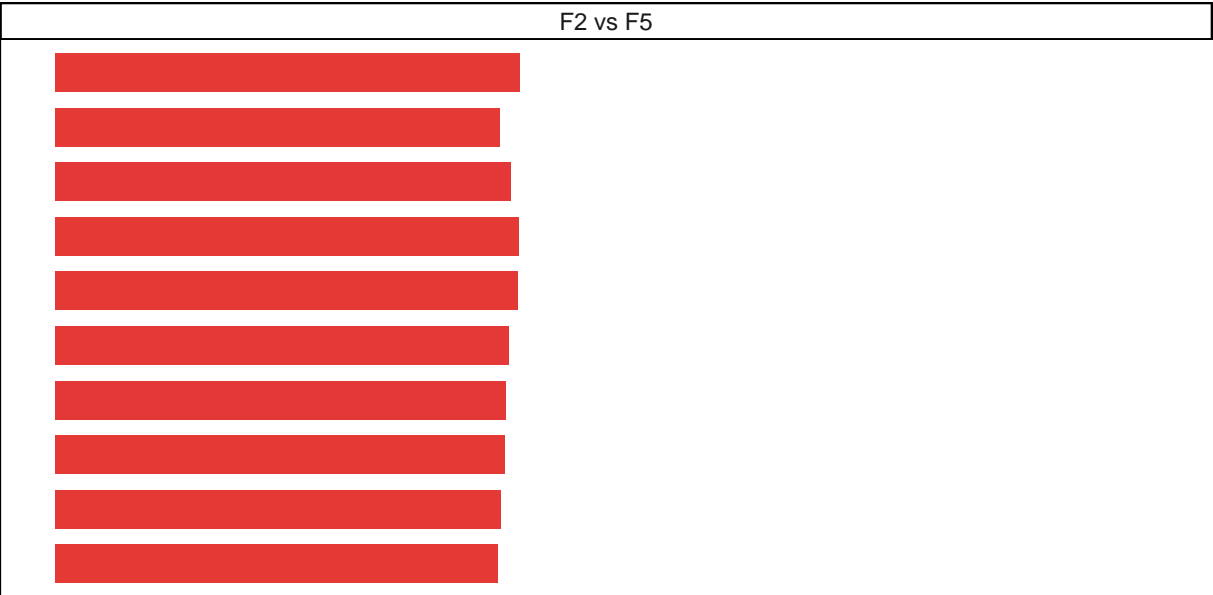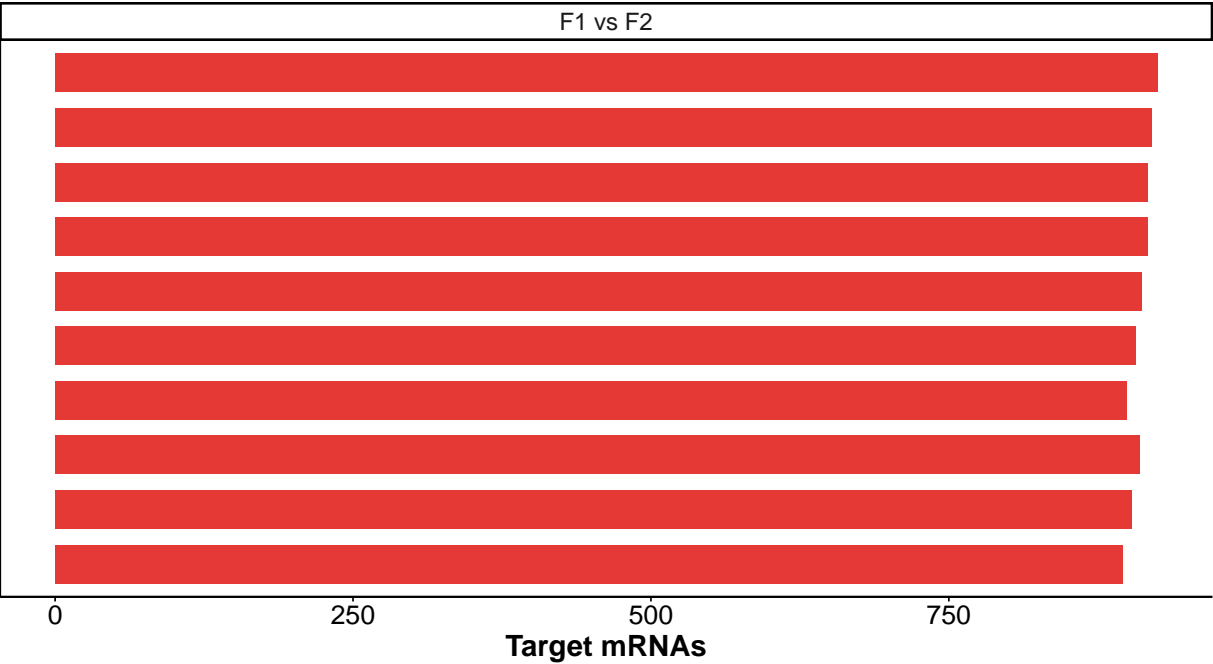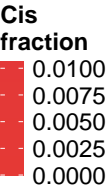

Supplement: Supplementary file 1 [file animals-16-01351-s001.zip › Figure S8_DEL_hub_network.pdf]

# Chromosomal distribution of cis DEL-mRNA pairs

F5 vs SYF F2 vs F5 F1 vs F2

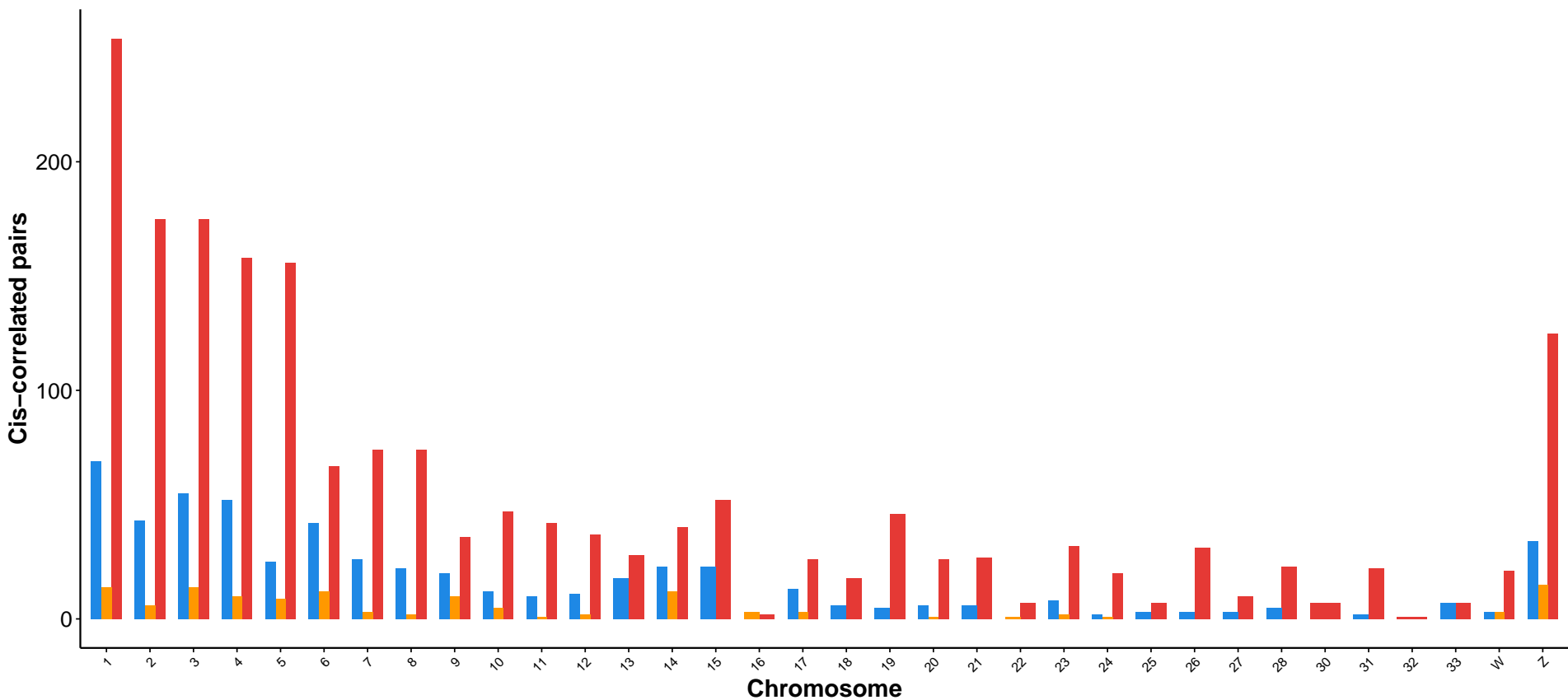

Supplement: Supplementary file 1 [file animals-16-01351-s001.zip › Figure S9_DEL_cis_chromosome.pdf]
